# ChatGPT: perspectives from human–computer interaction and psychology

**DOI:** 10.3389/frai.2024.1418869

**Published:** 2024-06-18

**Authors:** Jiaxi Liu

**Affiliations:** Wee Kim Wee School of Communication and Information, Nanyang Technological University, Singapore, Singapore

**Keywords:** ChatGPT, large language model, human–computer interaction, psychology, society

## Abstract

The release of GPT-4 has garnered widespread attention across various fields, signaling the impending widespread adoption and application of Large Language Models (LLMs). However, previous research has predominantly focused on the technical principles of ChatGPT and its social impact, overlooking its effects on human–computer interaction and user psychology. This paper explores the multifaceted impacts of ChatGPT on human–computer interaction, psychology, and society through a literature review. The author investigates ChatGPT’s technical foundation, including its Transformer architecture and RLHF (Reinforcement Learning from Human Feedback) process, enabling it to generate human-like responses. In terms of human–computer interaction, the author studies the significant improvements GPT models bring to conversational interfaces. The analysis extends to psychological impacts, weighing the potential of ChatGPT to mimic human empathy and support learning against the risks of reduced interpersonal connections. In the commercial and social domains, the paper discusses the applications of ChatGPT in customer service and social services, highlighting the improvements in efficiency and challenges such as privacy issues. Finally, the author offers predictions and recommendations for ChatGPT’s future development directions and its impact on social relationships.

## 1 Introduction

ChatGPT, developed by OpenAI, is a conversational system based on the large language model GPT (Generative Pre-trained Transformer). This product aims to achieve smooth, natural conversations with human users through natural language processing technology. ChatGPT has broad applications in customer service, educational tutoring, entertainment interactions, and more (Kocoń et al., 2023).

ChatGPT can understand complex queries and commands, producing fluid, coherent natural language responses. The system can remember conversation history and comprehend contextual information, thus providing accurate and relevant responses across multiple rounds of dialog (Nah et al., 2023). Additionally, it supports multiple languages, serving global users. ChatGPT not only understands and responds to factual questions but also simulates emotional interactions, offering a more humanized communication experience. By continuously learning from user feedback and dialog data, ChatGPT can self-optimize to enhance dialog quality and user experience.

ChatGPT has been widely applied in various fields, becoming a revolutionary tool. In customer service, it significantly improves response speed and efficiency by automatically answering common questions. In the education sector, ChatGPT acts as an intelligent tutoring assistant, offering personalized learning advice and materials to help students enhance their learning efficiency. Moreover, it assists in content creation, helping users write articles, reports, and creative writing. In entertainment and social media, ChatGPT generates creatively interactive content, bringing a new interactive experience to users. These application scenarios demonstrate ChatGPT’s powerful capabilities in understanding and generating natural language, as well as its enormous potential in improving human–computer interaction, boosting work efficiency, and enriching people’s lives.

The exploration of ChatGPT’s impacts has been extensive, yet existing research predominantly focuses on technical aspects and societal implications, leaving a notable gap in understanding its effects on human–computer interaction and user psychology. While prior studies have elucidated the technical architecture of ChatGPT and its broader societal implications, there remains a scarcity of research examining its nuanced effects on the dynamics of human–computer interaction and the psychological responses of users.

Based on the above background, this paper comprehensively analyzes the impact of ChatGPT in the fields of HCI, psychology, and society. Section 2 elaborates on the research purpose, detailing the research objectives and key research questions: How does ChatGPT impact human–computer interaction? What are the psychological effects of interacting with ChatGPT? Section 3, Methodology, describes the research methods employed, including data collection techniques and the inclusion and exclusion criteria used to ensure the relevance and quality of the selected studies. In Section 4, Technical Description, we explore the technical evolution of ChatGPT and its breakthroughs in the HCI field, highlighting its development history and the changes and impacts it has brought to natural language interaction. Section 5, Psychological Implications, examines the effects of ChatGPT on psychological support, emotional regulation, and social relationships, analyzing its influence on both human-to-human and human–machine interactions. Section 6, Social Implications, discusses the opportunities presented by ChatGPT in education, healthcare, and scientific research, as well as the challenges and risks such as the dissemination of false information, employment impact, and data privacy security. Section 7, Business Implications, addresses the impact of ChatGPT on various business domains. This section explores the opportunities and challenges in business environments such as intelligent customer service and digital marketing to understand the potential benefits and pitfalls of integrating ChatGPT into business operations. In Section 8, the Future Outlook discusses the directions for optimizing ChatGPT, including enhancing understanding and generation capabilities, multimodal interaction, and personalized language generation. This section also considers potential changes in social relationships and strategies to avoid social and ethical issues.

## 2 Research purpose

**Figure 1:**
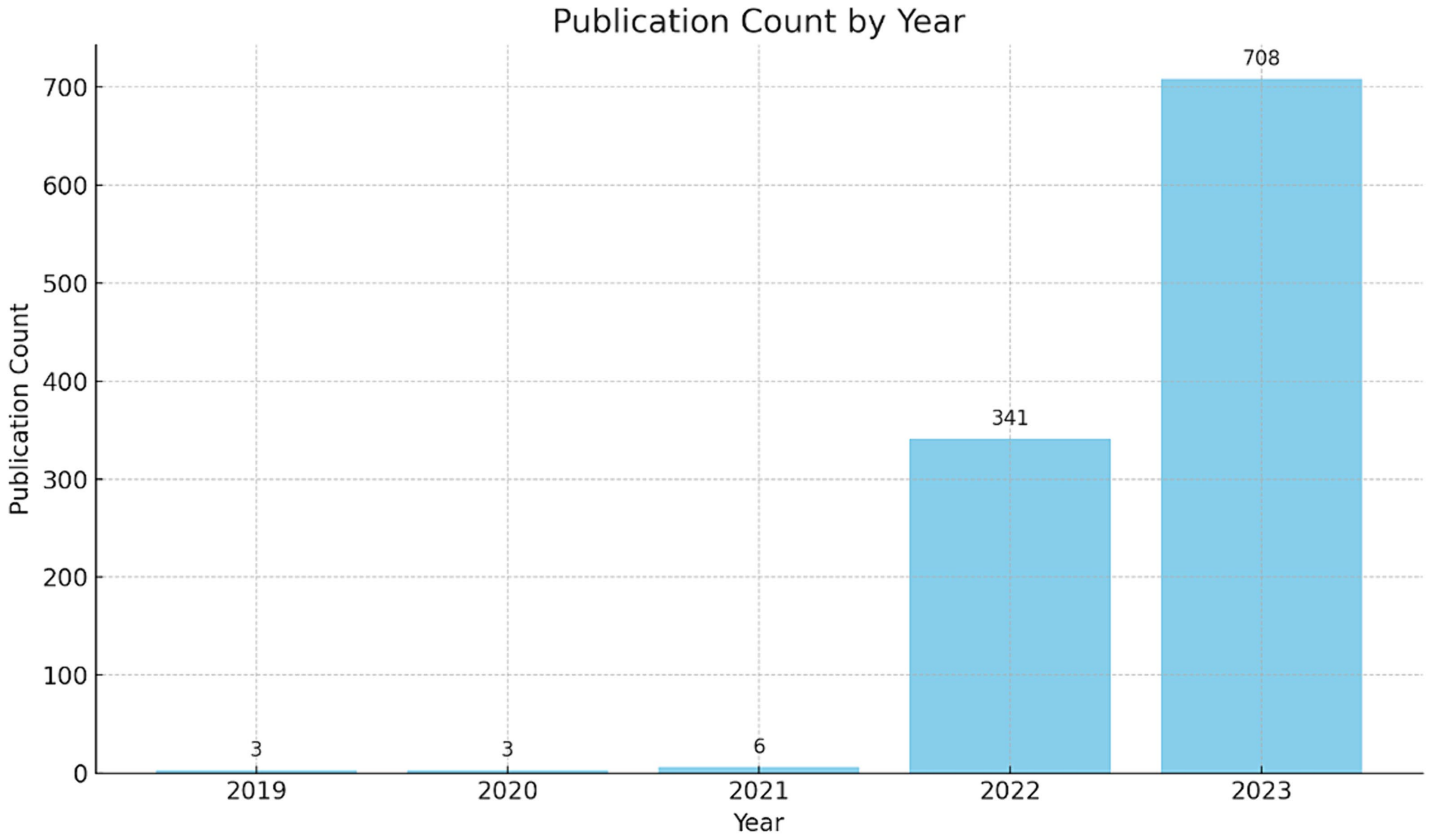
Annual statistics related to ChatGPT publications.

Figure 1 shows the annual publication trends of papers related to ChatGPT (obtained from Google Scholar with titles containing “ChatGPT”). From 2019 to 2023, the research output has grown exponentially. In 2022, the number of publications surged dramatically to 341 papers, and further increased to 708 papers in 2023. This trend indicates a rapid expansion of research activities driven by technological advancements and heightened interest from both academia and industry (Liu et al., 2023). The substantial growth in 2022 and 2023 highlights the dynamic and evolving nature of research in this field.

**Figure 2:**
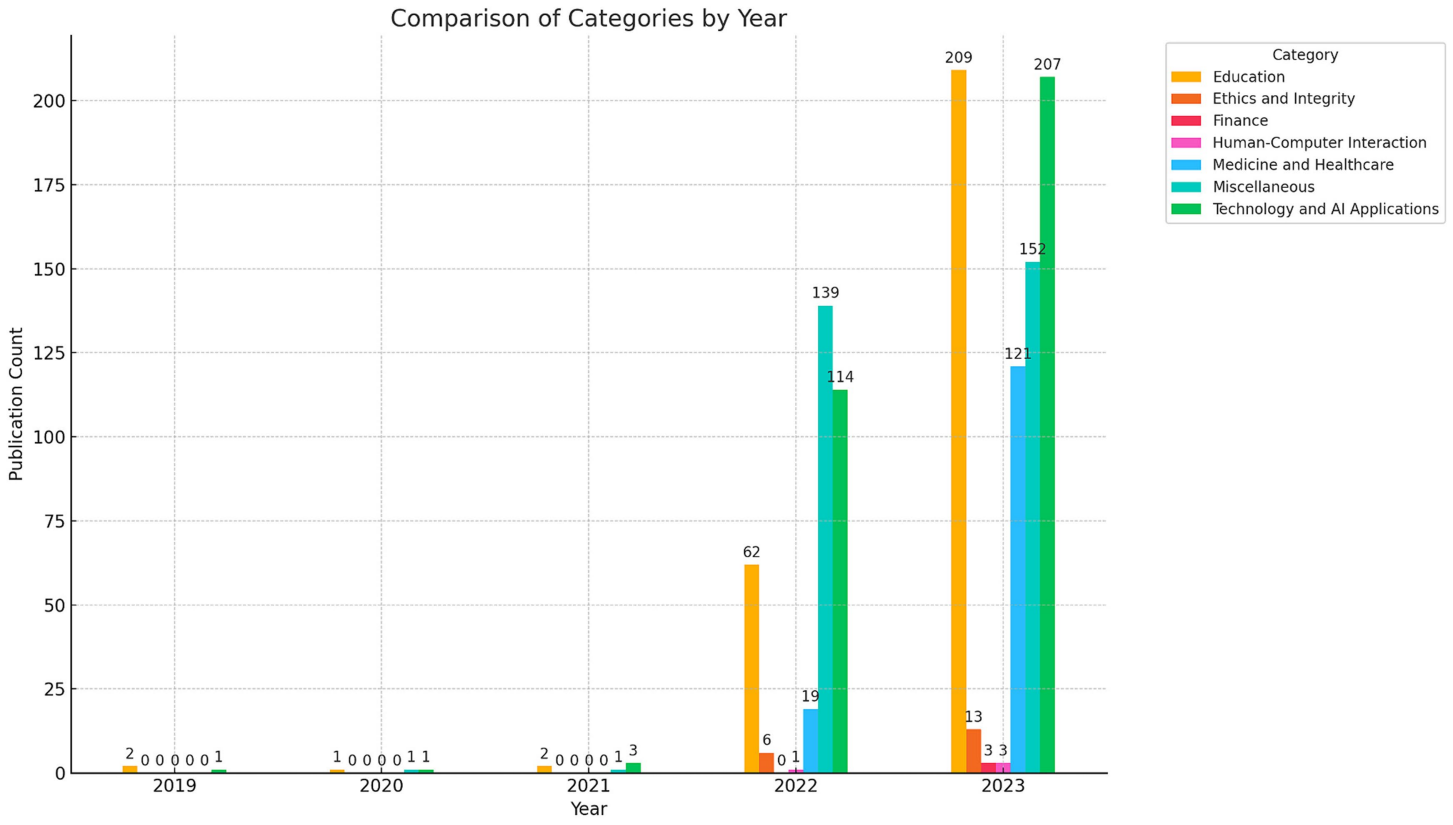
Categorical statistics for ChatGPT Publications.

Figure 2 categorizes the research results of recent years by different fields. From 2019 to 2023, there is a significant increase in research output in all fields. Initially, the number of published papers is low, with minimal contributions in the various fields. However, from 2022 onwards, there is a significant increase in research activity, with substantial growth in research outputs in categories such as education, technology and healthcare (Nah et al., 2023). By 2023, the scope of research in areas such as Ethics and Integrity and Finance is also gradually expanding (Farhat et al., 2024). Despite this overall growth, it is worth noting that research focusing on human–computer interaction and its psychological impact remains relatively limited, suggesting that this area could benefit from more academic attention and exploration.

Additionally, as shown in Table 1, the author selects 12 comprehensive review papers from numerous studies and conducted a horizontal comparison.

**Table 1:** Comparison of core points in review papers.

Title	Authors/Published date	Methodology	Core points	Characteristics
A survey on ChatGPT: AI–generated contents, challenges, and solutions	Wang et al. (2023)	Literature review	Examines AI-generated contents created by ChatGPT. Discusses the challenges and solutions related to its use. Provides insights into the potential applications and ethical considerations.	Comprehensive overview of content and challenges.
Exploring ChatGPT capabilities and limitations: a survey	Koubaa et al. (2023)	Survey	Investigates the capabilities of ChatGPT. Highlights the limitations and potential areas of improvement. Discusses its performance across various tasks and domains.	Detailed assessment of capabilities and limitations.
ChatGPT: jack of all trades, master of none	Kocoń et al. (2023)	Literature review	Analyzes the versatility of ChatGPT in handling diverse tasks. Critically evaluates its performance, suggesting that it is a generalist rather than a specialist. Proposes areas for enhancing its specialized capabilities.	Critical evaluation of versatility and specialization.
ChatGPT: a comprehensive review on background, applications, key challenges, bias, ethics, limitations, and future scope	Ray (2023)	Literature review	Provides an extensive review of ChatGPT’s development and underlying technology. Discusses various applications in different sectors. Addresses the key challenges, including biases and ethical concerns. Suggests future directions for research and development.	Extensive coverage of development, applications, and challenges.
A comprehensive survey of ChatGPT: advancements, applications, prospects, and challenges	Nazir and Wang (2023)	Literature review	Reviews the advancements made in the development of ChatGPT. Explores its applications and potential future impacts. Discusses the challenges faced in integrating ChatGPT into various fields.	Thorough review of advancements and applications.
Summary of ChatGPT-related research and perspective toward the future of large language models	Liu et al. (2023)	Literature review, research perspective	Summarizes existing research on ChatGPT. Provides a perspective on the future development of large language models. Highlights the implications of ChatGPT for future AI research.	Summary of current research and future perspectives.
Generative AI and ChatGPT: applications, challenges, and AI-human collaboration	Nah et al. (2023)	Case studies	Examines the applications of generative AI, with a focus on ChatGPT. Discusses the challenges of AI-human collaboration. Proposes strategies for effective integration of ChatGPT into collaborative environments.	Examination of applications and collaboration challenges.
ChatGPT: a brief narrative review	Gupta et al. (2023)	Narrative review	Provides a brief review of ChatGPT’s capabilities and applications. Discusses its limitations and potential improvements. Highlights key areas where ChatGPT has been effectively utilized.	Concise overview of capabilities and applications.
The social impact of generative AI: an analysis on ChatGPT	Baldassarre et al. (2023)	Qualitative analysis, social impact study	Analyzes the social impact of generative AI, focusing on ChatGPT. Discusses how ChatGPT affects social interactions and communication. Addresses the ethical and societal implications of widespread ChatGPT use.	Analysis of social impact and ethical implications.
The scholarly footprint of ChatGPT: a bibliometric analysis of the early outbreak phase	Farhat et al. (2024)	Bibliometric analysis, quantitative analysis	Provides a bibliometric analysis of the early research on ChatGPT. Examines publication trends, citation patterns, and collaborative networks. Identifies emerging research areas and future directions.	Bibliometric analysis of early research trends.
ChatGPT and Open-AI models: a preliminary review	Roumeliotis and Tselikas (2023)	Scoping review, literature review	Offers an overview of the training process and fundamental functionality of ChatGPT. Presents the first comprehensive literature review of this technology. Discusses potential implications on existing knowledge and technology.	Preliminary review of training and functionality.
The beginning of ChatGPT – a systematic and bibliometric review of the literature	Baber et al. (2023)	Systematic review, bibliometric analysis	Provides a systematic and bibliometric review of the early literature on ChatGPT. Highlights research trends and key findings in the initial studies on ChatGPT. Discusses the scope and direction of future research on ChatGPT.	Systematic and bibliometric review of early literature.

Despite extensive research on the technical capabilities and applications of ChatGPT, there is still a significant gap in understanding its impact on human–computer interaction and user psychology (Elyoseph and Levkovich, 2023). Existing research has largely ignored how interaction with ChatGPT affects users’ emotional state, mental health, and overall well-being (Salah et al., 2023). In particular, there is limited research on how ChatGPT affects users’ short-term emotional responses and long-term psychological well-being in different contexts (Liu and Sundar, 2018). In addition, behavioral changes that may result from the use of ChatGPT, such as increased reliance on AI for decision-making and altered patterns of social interaction, have not been explored in depth (Rad and Rad, 2023).

Addressing these issues is critical to the development of more empathetic, trustworthy, and user-centered AI technologies (Hohenstein et al., 2023). Understanding the impact of ChatGPT on user psychology and behavior can help optimize its design to better meet user needs and ensure that it does not adversely affect users.

Given these significant gaps, it is critical to fully explore the impact of ChatGPT on HCI and user psychology. By addressing these unexplored areas, the proposed research will contribute to a deeper understanding of the psychological and interactive dimensions of ChatGPT (Hohenstein et al., 2023). This exploration will provide valuable insights into the potential benefits and risks of widespread adoption of large-scale language modeling, thereby enhancing the knowledge base of the field and informing future AI technology development.

In addition, we will investigate the application of ChatGPT in business and social domains. This will not only highlight the advantages of ChatGPT in real-world applications, but also identify and address the challenges it faces in practice, thereby promoting its wider and more effective use.

Therefore, we propose the following research questions:

*RQ1*: What are the technical principles of ChatGPT and its breakthrough on human–computer interaction?

*RQ2*: What are the psychological and societal impacts of ChatGPT on users?

*RQ3*: How is ChatGPT applied in the commercial and social domains? What challenges exist?

*RQ4*: What predictions and recommendations can be made for the future development of ChatGPT and its impact on social relationships?

## 3 Methodology

### 3.1 Inclusion and exclusion criteria

The paper must focus on ChatGPT or similar large language models and include perspectives related to psychology or HCI. Articles must clearly state their research objectives and questions, particularly those related to HCI and psychology, where the research purpose should be related to user experience, interaction design, or psychological impact. During the full-text screening phase, articles published before 2022 and those that do not explicitly use the terms “ChatGPT,” “GPT,” or equivalent terms for large language models are excluded. By applying this criterion, the current study ensures data accuracy and timeliness. Additionally, the selected papers are exclusively in English, excluding studies primarily focused on specific regions without broad applicability or dissemination value.

### 3.2 Search strategy

The development of the search strategy included multiple term combinations to ensure the capture of relevant literature on various aspects of ChatGPT. Terms like “GPT” or “LLM” must be present. Additionally, the search included related synonyms and terms such as “natural language processing” (NLP), “conversational agents,” and “artificial intelligence” (AI) to capture more relevant studies.

### 3.3 Data sources

The article searched the following databases: ACM Digital Library, IEEE Xplore, Scopus, and Google scholar. These databases cover the intersection of chatbots, artificial intelligence, natural language processing, and psychology. References were obtained from all papers identified through relevant reviews and searches. Additionally, the impact factors of journals in Web of Science were checked, focusing mainly on published papers with an impact factor above 3.0. During the screening, the authors’ academic background, affiliations, and research areas were reviewed to ensure relevance to the published content.

## 4 Technical description

### 4.1 Technical evolution

**Figure 3:**
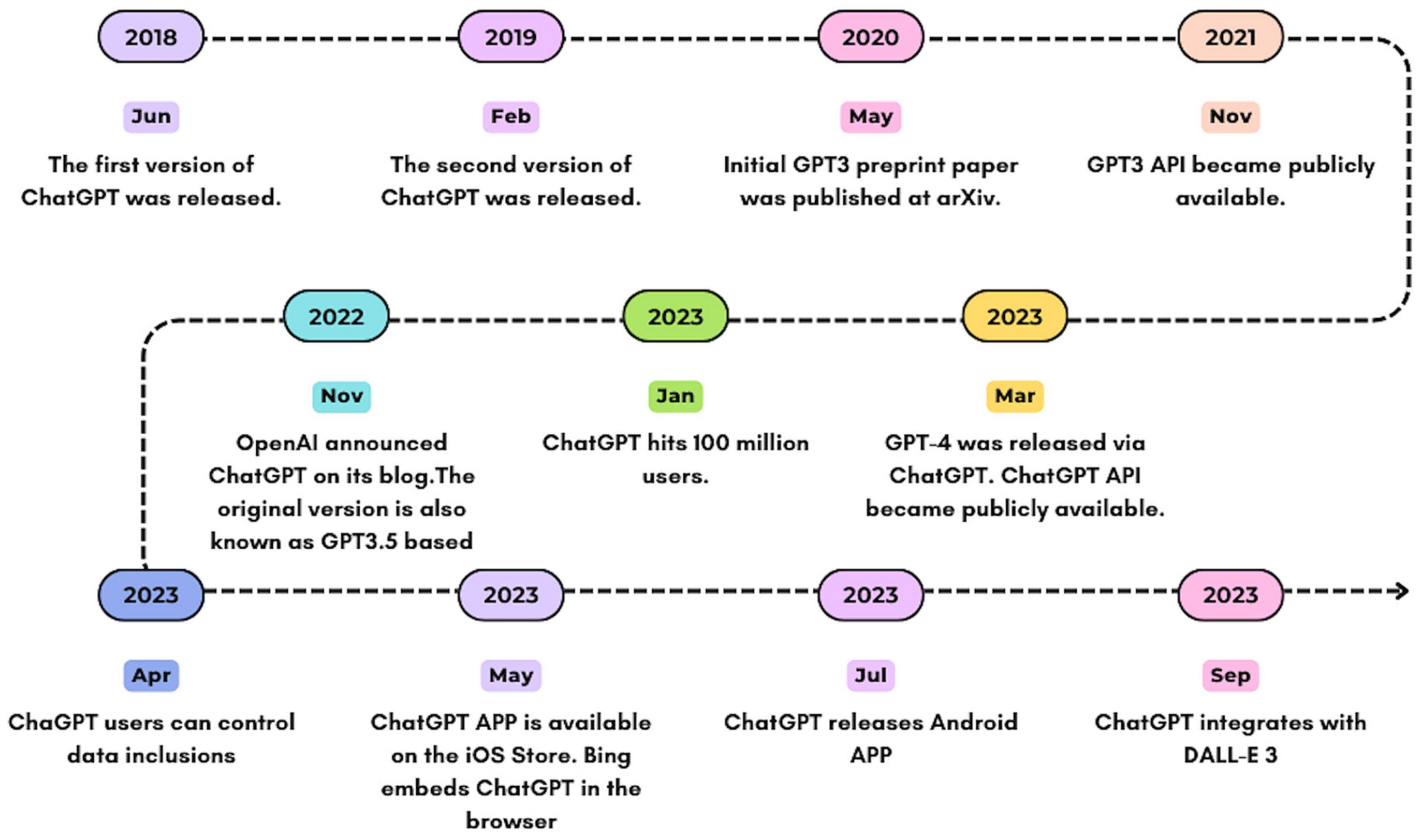
Brief History of ChatGPT.

On November 30, 2022, OpenAI unveiled ChatGPT with a demonstration that quickly captured the public’s imagination. Soon, users began sharing its myriad capabilities, from trip planning and fable writing to programming. Its rapid rise to popularity saw it amass over a million users in just five days (Marr, 2023).Within five days, the chatbot attracted more than a million users (Marr, 2023). ChatGPT was born out of GPT3.5, and the GPT model has gone through several iterations before being released to the public. Figure 3 shows a brief history of ChatGPT.

The journey began with the introduction of GPT-1 in June 2018, marking the inception of the Generative Pre-trained Transformer series. This model was composed of 117 million parameters, establishing the foundational framework for ChatGPT (Ali, 2023). GPT-1 showcased the efficiency of unsupervised learning for language tasks by training on a dataset of books to anticipate subsequent words within sentences (Ali, 2023).

GPT-2 was released in February 2019. This version achieved a substantial enhancement with its 1.5 billion parameters (OpenAI, 2019). It displayed significant strides in text generation and the ability to produce coherent texts spanning multiple paragraphs. But GPT-2 was not initially released to the public because of its potential for abuse. After OpenAI rolled out in phases to study and mitigate potential risks, the model was finally launched in November 2019.

The release of GPT-3 in June 2020 represented a monumental leap, trained on an unprecedented 175 billion parameters (Nazir and Wang, 2023). Its sophisticated text generation capabilities have found broad application, ranging from composing emails and articles to generating poetry and programming code, alongside its prowess in answering factual queries and performing language translation. ChatGPT, as it is known today, evolved from GPT-3.

The latest version of GPT-4 continues this trend of exponential improvement. More adequate parameter training improves the model performance of GPT-4 and enables it to have more accurate text capabilities. It can accept images and text input, and send text output. Although ChatGPT is less capable than humans in many real-world scenarios, it demonstrates human-level performance on a variety of professional and academic benchmarks (OpenAI, 2023). Table 2 shows a comparison of the capabilities of each version of GPT.

**Table 2:** Comparison of capabilities of various GPT versions.

Name	Release time	Model architecture	Parameter quantity	Dataset	Ability	Highlights
GPT	2018	Transformer	110 million	WebText, the web page data pointed to by the Reddit link is filtered and obtained	Understand and generate language Versatility	The introduction of Transformer architecture
GPT-2	2019	Transformer	1.5 billion	Compared to GPT-1, a larger WebText data set is used	Improved text generation capabilities Complete tasks such as translation, Q&A, and summarization even without training for a specific task	Powerful adaptability without task-specific training Naturalness and coherence of text generation
GPT-3	2020	Transformer	175 billion	Including Common Crawl, WebText2, Books1, Books2 and Wikipedia, it is one of the largest language model data sets to date	Ability to adapt and perform new tasks when provided with only a few examples High quality text generation	Few-Shot Learning High quality and fidelity of text generation
GPT-3.5	2022	Transformer	176 billion	Common Crawl, WebText2, contains text crawled from the Internet, Books1 and Books2, Wikipedia	Improved accuracy when processing complex dialog and text	Optimization and improvements in details
GPT-4	2023	Transformer, may include new optimizations	1.76 trillion	Supervised learning on a large dataset, then reinforcement learning using both human and AI feedback	Generate pictures from text Document parsing More powerful language generation and semantic understanding capabilities	Multimodal interaction Personalized service

### 4.2 Training models

#### 4.2.1 Transformer model: the cornerstone of GPT

As a large language model, ChatGPT inevitably utilizes neural network algorithms. The Transformer model used by ChatGPT is a revolutionary deep learning architecture first introduced in the paper “Attention is All You Need” by Vaswani et al. (2017). It is primarily designed for sequence-to-sequence tasks, such as text translation, text generation, and other applications in natural language processing. Through its unique attention mechanism, the Transformer model can efficiently process long-distance dependencies and has shown significant improvements in training efficiency and effectiveness (Wu et al., 2023).

In ChatGPT, these characteristics of the Transformer model are utilized to understand and generate human language. Through massive pre-training, the model learns the language patterns and knowledge in vast language datasets, enabling it to generate coherent, realistic text (Wu et al., 2023). ChatGPT leverages the capabilities of the Transformer not only to produce high-quality text but also to understand complex queries, provide useful information, and even simulate conversations.

#### 4.2.2 Human feedback reinforcement learning (RLHF)

LLMs are generally functional after pre-training. But ChatGPT also went through another pioneering OpenAI process called Reinforcement Learning from Human Feedback (Ouyang et al., 2022). This model worked in several stages.

##### 4.2.2.1 Pre-training

The model is initially pre-trained on a large dataset to learn the basic structure and knowledge of language. This stage uses unsupervised learning methods, aiming to equip the model with broad language understanding and generation capabilities. In the RLHF framework, the pre-trained model is usually based on the Transformer architecture, which provides the foundation for the pre-training stage (Zhao et al., 2023).

##### 4.2.2.2 Human feedback collection

At this stage, human evaluators interact with the model or assess the texts generated by the model. Evaluators provide positive or negative feedback based on the quality, relevance, and accuracy of the texts. This feedback data is used to guide further learning of the model. The machine uses the collected human feedback to train a new model, learning to differentiate between good and bad outputs. This step is achieved by comparing the preference order of different text outputs, aiming to make the model understand the preference criteria of human evaluators (Rafailov et al., 2023).

##### 4.2.2.3 Reinforcement learning

**Figure 4:**
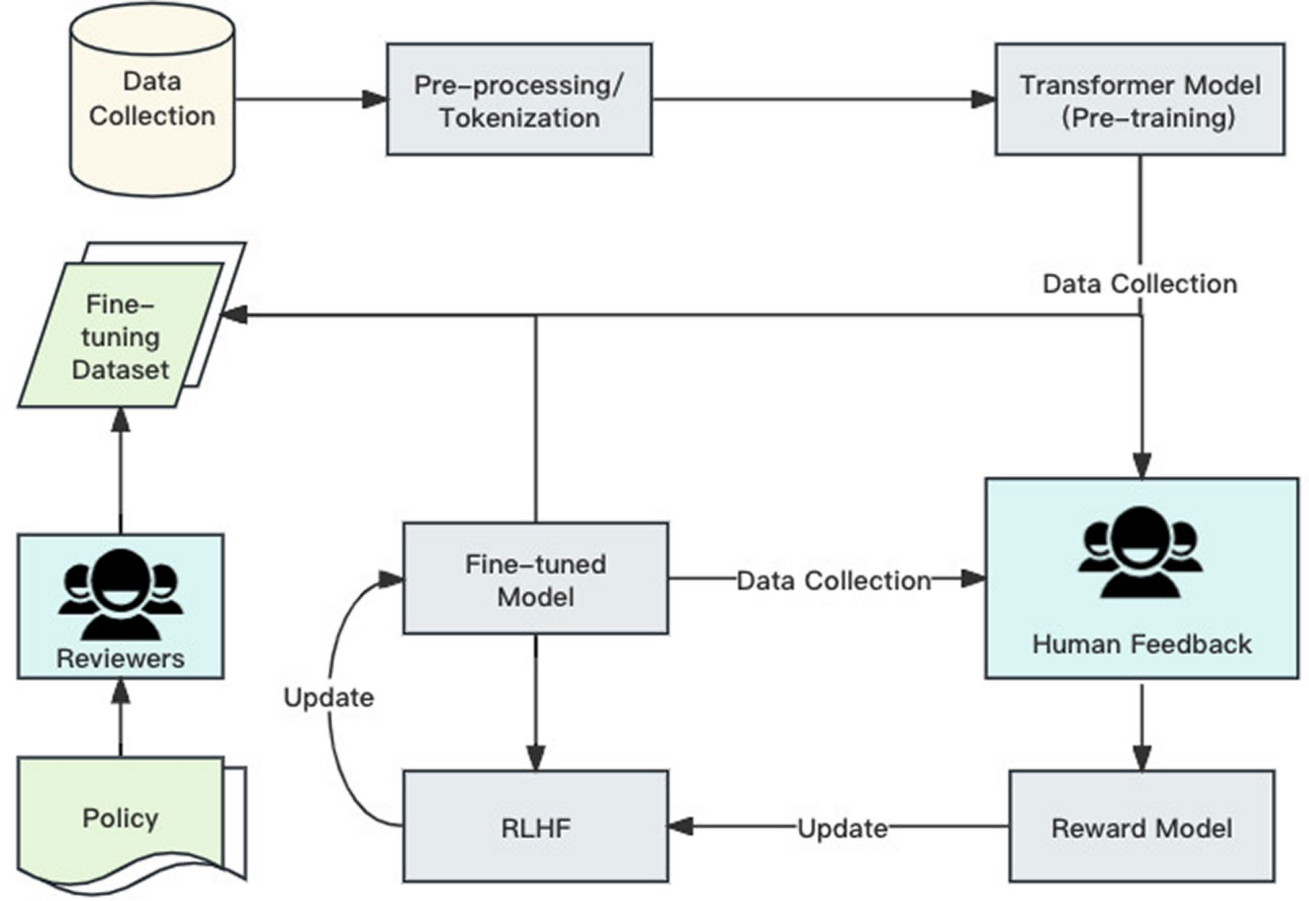
ChatGPT training process.

Figure 4 shows the overall training process of ChatGPT. By using the RLHF model, ChatGPT can obtain direct feedback from humans, thereby generating more relevant and accurate texts; at the same time, the model can effectively filter harmful or incorrect texts, as human evaluators mark these contents in the feedback. This approach also allows the model to adjust according to specific needs and standards, which can improve the adaptability and flexibility of the model.

### 4.3 Breakthrough on HCI

#### 4.3.1 Challenges in achieving natural human–computer dialog

Although human communication follows specific patterns, it is also highly ambiguous in a linguistic context, filled with emotions, empathy, and implied meanings. In real life, Sarcasm and metaphors are often applied in specific scenarios, and social norms and culture can also influence the meaning of language.

Developing humanized human–computer dialog systems faces several significant challenges. A core challenge is accurately understanding complex and ambiguous human language. Humans often use context-dependent language, and the meanings of our words can change based on the situation. Computers need to understand the context to make appropriate responses, making it difficult for machines to grasp the true meaning of what people say. Another challenge is that human language often conveys emotions and emotional states, requiring interlocutors to discern different emotions and respond accordingly. A sufficiently natural dialog also requires the machine to provide emotional feedback, which is particularly challenging for machines. The third challenge is basic common sense and knowledge reserves. High-quality communication requires interlocutors to have sufficient common sense and general knowledge, otherwise, they cannot provide reasonable answers. The final challenge is how machines can accurately identify and correct misunderstandings or errors. This requires machines to have self-learning and adaptation capabilities, thus to recover from mistakes and optimize strategies.

#### 4.3.2 Breakthroughs in NLP interactions with ChatGPT

**Figure 5:**
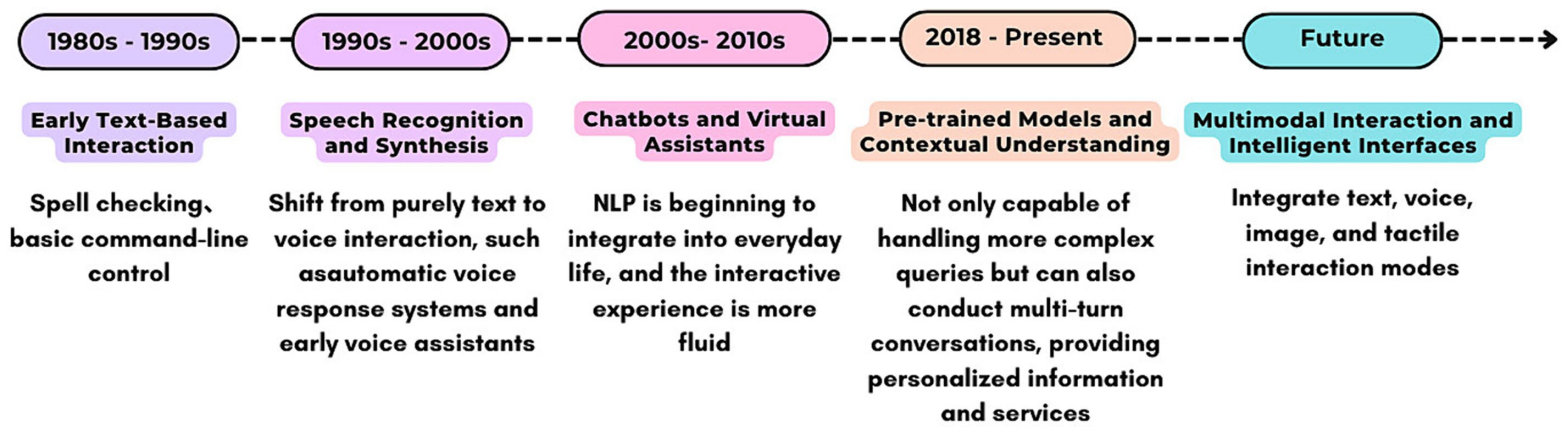
NLP development timeline.

The application and development of Natural Language Processing (NLP) in the Human–Computer Interaction (HCI) field have gone through several important stages, continuously driving the innovation of human–computer interaction methods. As shown in Figure 5, the first generation of NLP and AI technology was not mature, resulting in human–computer communication systems that were inhuman and difficult to use directly for users (Jones, 1994). In the second and third generations of NLP technology, natural language understanding and natural language generation capabilities continued to develop, enabling virtual assistants to provide a smoother and more natural interaction experience (Khurana et al., 2022). However, NLP at this stage struggled to understand complex contextual content, and Chatbots could not help humans intuitively perform complex operations or obtain the needed information. Expectations for chatbots were lower at this stage. For example, research by Mozafari et al. (2020) showed that disclosing its robot identity when customer service gives incorrect answers could lead to higher service satisfaction. This is because users have lower expectations for robot services. Now, ChatGPT, representing the fourth generation of human–computer interaction technology, can not only better understand user intentions and context, generate more accurate, natural replies, but also handle complex tasks and provide personalized services. This means that anthropomorphic features of robots may be more widely accepted and used in the future, and collaboration between humans and machines will become more seamless and efficient.

The Transformer model, RLHF model, etc., mentioned earlier, all contribute to the development of ChatGPT’s powerful language understanding and generation capabilities. They endow ChatGPT with the following powers to achieve general humanoid dialog.

##### 4.3.2.1 Massive knowledge reserves

As mentioned earlier, ChatGPT has been trained on a large and diverse dataset, acting like an encyclopedia in the cloud; it can understand users’ queries on multiple topics and provide relatively accurate analysis. Regardless of who talks to ChatGPT, it can effectively answer general questions. According to data from OpenAI (2023), GPT-4 has passed the unified bar exam, law school admission test, SAT, graduate entrance exams, etc., and achieved impressive results.

##### 4.3.2.2 Rich vocabulary

**Figure 6:**
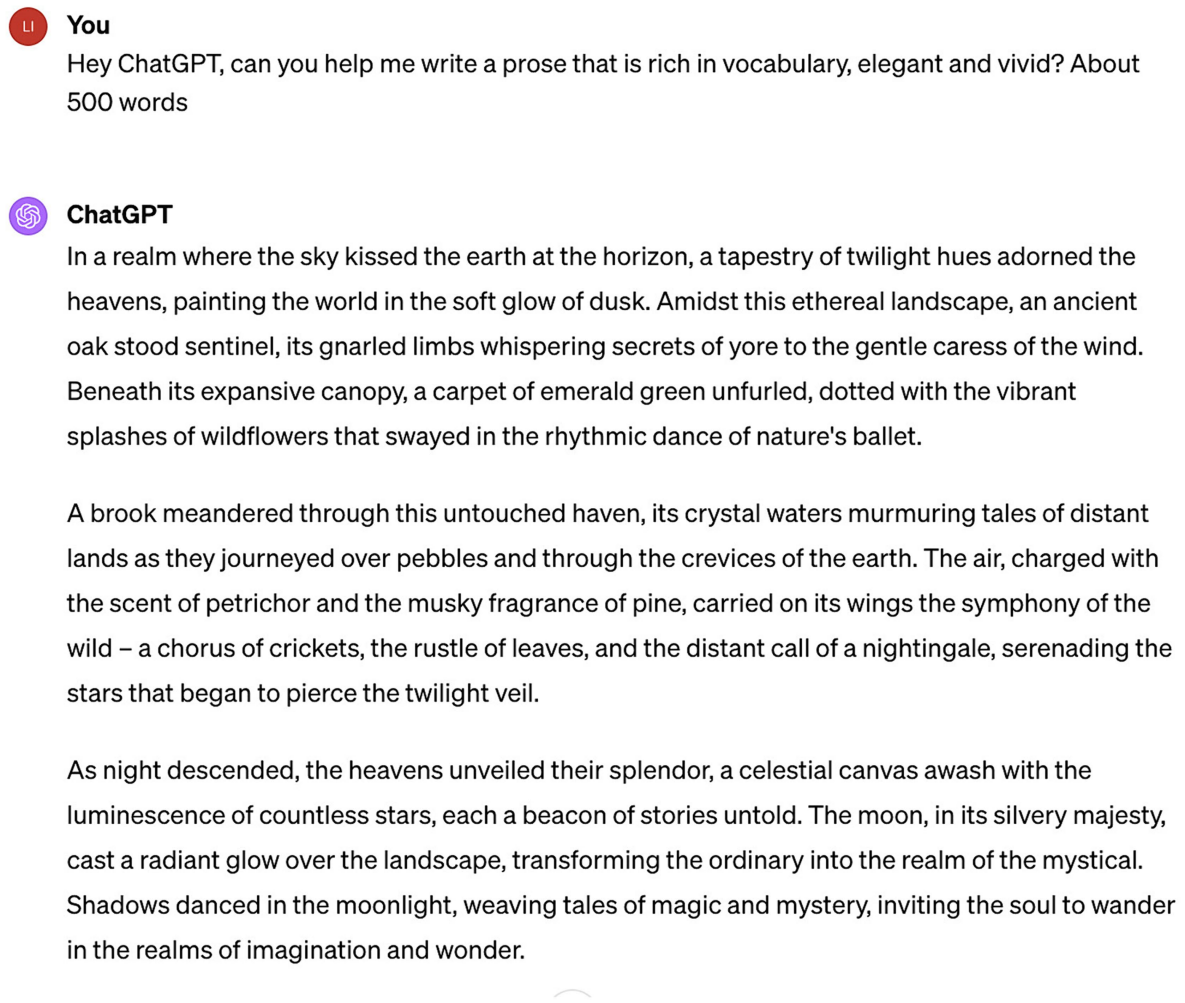
Rich vocabulary expression of ChatGPT.

Utilizing an extensive database of textual information, ChatGPT is equipped with a vast lexicon, enabling it to offer comprehensive insights across a myriad of subjects. This enriches interactions and adds a layer of humanity to conversations, allowing users to conduct more intricate inquiries and receive thorough, insightful responses. Such capabilities not only extend the interaction time between humans and machines but also facilitate deeper dialog, since users are more likely to explore a series of subsequent questions based on the responses provided by the machine. Kaplan (2024) highlights that GPT-3 encompasses a vocabulary of approximately 14,735,746 words, far surpassing the lexical range of the average human. While not every word is equally pertinent across all contexts, ChatGPT prioritizes certain terms more frequently. As shown in Figure 6, the author engaged in multiple conversations with ChatGPT, verifying that ChatGPT’s vocabulary capabilities greatly improved the attractiveness and approachability of the conversations.

##### 4.3.2.3 Deeper understanding of language

ChatGPT has the capability to accurately grasp the nuances of language, contextual cues, and scenarios, thereby offering users responses that feel more personalized and human-like. The nature of human language often embodies ambiguity, where a single expression or sentence might unfold into various interpretations. ChatGPT adeptly navigates through this ambiguity, formulating responses that encompass different potential meanings of a phrase (Cai et al., 2023). For instance, when “haha” is entered into ChatGPT, it’ll remind you that, beyond signifying amusement or happiness, this expression could also hint at slight displeasure or irony within the Chinese context. In dialogs spanning multiple turns, ChatGPT is proficient in automatically pinpointing the subjects of pronouns. Say a user talks about their friend Cathy; ChatGPT can discern that subsequent mentions of “she” indeed refer to Cathy.

**Figure 7:**
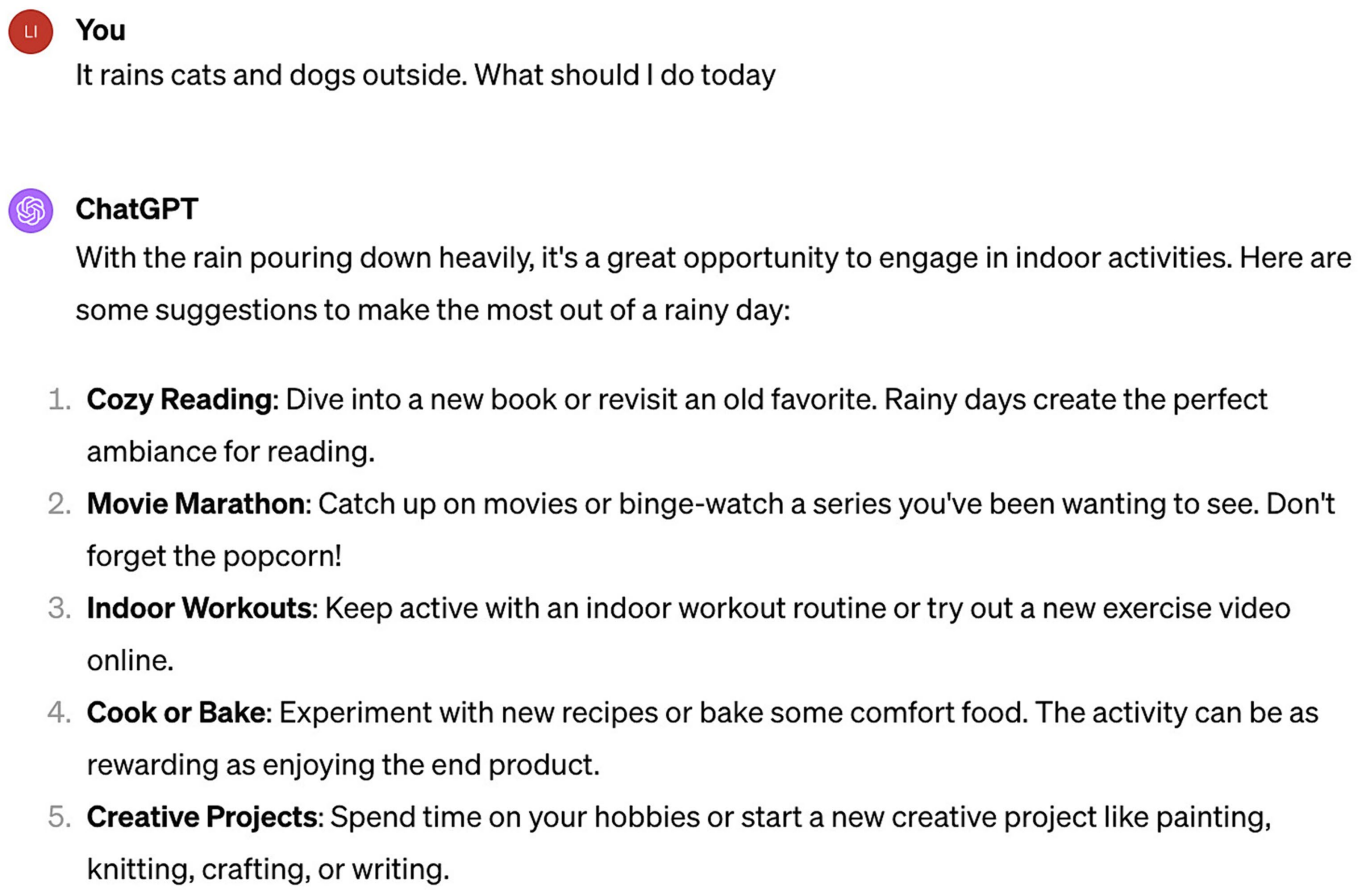
ChatGPT’s understanding of metaphors.

Moreover, ChatGPT possesses the ability to understand idiomatic and metaphorical language. Despite the intricate underlying meanings of idioms and metaphors, ChatGPT recognizes these figures of speech and crafts responses that reflect an understanding of their implied meanings. For example, as depicted in Figure 7, when the author sends “It rains cats and dogs,” ChatGPT comprehends the phrase as describing a downpour, not an actual fall of cats and dogs from the heavens.

##### 4.3.2.4 Understanding the context of conversation

**Figure 8:**
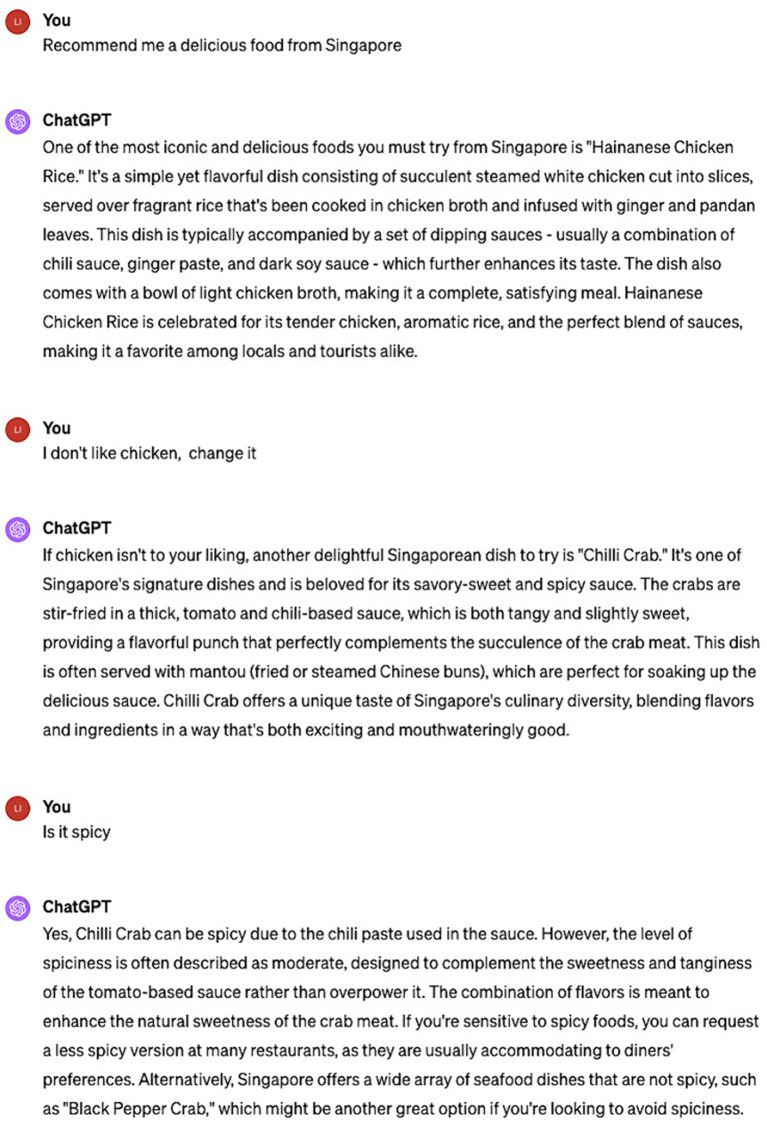
ChatGPT can better respond based on context.

ChatGPT adeptly tailors its language and responses to fit the context of the interaction. It has the capacity to grasp and recall previously established context, delivering more insightful and tailored responses. As illustrated in Figure 8, ChatGPT grasps the conversation’s context and adeptly builds on it with each exchange. By remembering the previous question and understanding the topic of the conversation, it provides a more relevant response. This skill in maintaining context and understanding the progression of a conversation allows ChatGPT to engage in communication that is informed by the background, mirroring human-like intelligence, personability, and fluidity.

##### 4.3.2.5 Emotional expression

**Figure 9:**
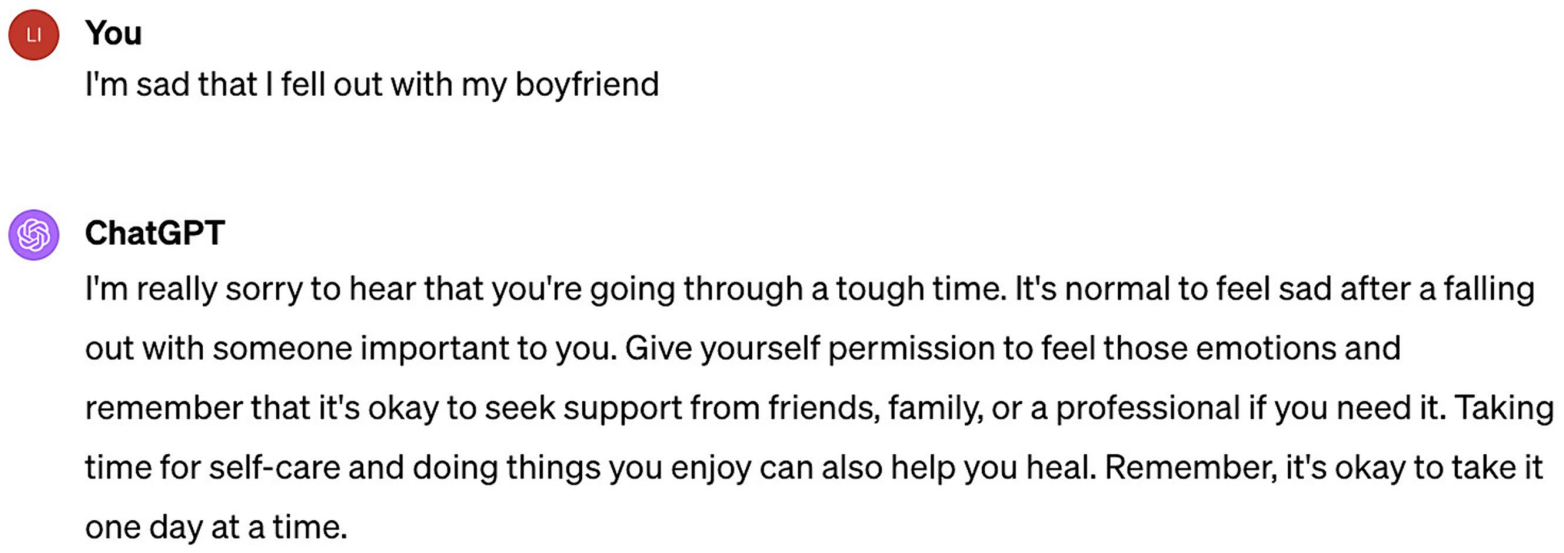
ChatGPT can bring emotional value to users.

The development of ChatGPT encompasses the ability to express and interpret emotions, akin to human interaction. ChatGPT is equipped to identify expressions of negative feelings and offer empathetic responses. Illustrated in Figure 9, when a user conveys distressing news to ChatGPT, it responds with compassion, assisting the user in navigating through their distress. In the same vein, ChatGPT can respond to positive emotional expressions like joy or enthusiasm. For instance, upon hearing a user announce a job promotion, ChatGPT might respond with, “Congratulations! That’s fantastic news.” ChatGPT extends beyond mere responses, actively offering emotional support where appropriate. For example, when the author talks about purchasing a book, ChatGPT commends the action, saying, “That’s excellent! Reading is a great avenue for acquiring knowledge and expanding your view of the world.”

#### 4.3.3 Comparison of ChatGPT’s conversational interaction with GUI

ChatGPT employs a conversational interface, i.e., a Natural Language Interface (NLI). As shown in Table 3, this type of interaction interface is distinct from Graphic User Interfaces (GUI), with each having its characteristics and advantages in terms of human-computer interaction methods, user experience, and applicable scenarios (Kumamoto and Ohta, 2004; Shah and Pareek, 2022).

**Table 3:** Comparison of NLI and GUI interactive interfaces.

Interface type	Interactive mode	User threshold	Discoverability	Task efficiency	Applicable scene	Error handling and feedback
NLI	Users communicate with cloud computing through natural language, which can be typing or voice. This type of interaction is closer to natural communication between humans.	It usually has a lower threshold for use because users can express their needs using the language they are familiar with without having to learn specific operating methods.	Features are less discoverable, and users may not know which commands they can use or what types of requests they can make unless the system provides prompts or help.	For complex queries or instructions, NLI may be more efficient because users can express requirements directly without having to navigate through multiple steps.	It is particularly suitable for use in environments that require rapid cloud computing or where there are no graphic display devices, such as smart speakers or other IoT devices.	Error handling can be more challenging because the system needs to understand multiple expressions and intentions. Providing accurate and helpful feedback is equally important.
GUI	Rely on visual elements such as buttons, icons, menus, and windows. Users interact with the application through operations such as clicking and dragging.	While intuitive and easy to use, for apps with complex functionality, users may need to spend time learning how to navigate and use different features.	Layout and visual cues make it easier for users to discover and understand available features and actions.	For intuitive or visually related tasks, a GUI may provide faster task completion because users can quickly identify icons and perform actions.	Ideal for applications that require rich interaction and high visualization, such as graphic design software, video games, and data visualization tools.	Through interface elements and instant visual feedback, users can immediately understand the results of operations or error messages.

ChatGPT Interface (Conversational Interface) versus Traditional Graphical User Interfaces (GUI) offers unique advantages in several aspects, especially in providing flexible user interactions, lowering usage barriers, and enhancing user experience. However, despite the many advantages provided by the ChatGPT interface, traditional GUIs may perform better in task efficiency and precise control due to their intuitive visual elements and structured layout. Therefore, the choice between interfaces depends on the application scenario, user needs, and personal preferences. In the future, we may see more hybrid interfaces combining ChatGPT with GUI to fully leverage the strengths of both.

## 5 Psychological implications

ChatGPT could have various psychological impacts on users, including both positive and negative effects.

### 5.1 Psychological support

Research indicates that chatbots are capable of significantly reducing anxiety and depressive symptoms (Fitzpatrick et al., 2017). Alanezi’s (2024) qualitative interviews revealed that nearly 80% of respondents found ChatGPT beneficial for managing symptoms of psychological distress by facilitating relaxation, mindfulness, and stress alleviation techniques usable in everyday life. Over half the participants felt that ChatGPT created an inclusive environment where individuals could openly share their emotions and concerns, receiving empathetic replies that made them feel acknowledged and validated. One interviewee from Alanezi’s (2024) study noted:

*I said the application that I am sad, and its reply that it was sorry to hear that I am sad, and provided some suggestions to improve my mood…These things made me feel more happy seeing that a machine is caring for me, and it was doing its best for making me feel happy, like it said a joke…*

Chatbots, while beneficial, can sometimes worsen mental health conditions, particularly if they fall short in providing adequate support or guidance. This inadequacy can lead to user frustration or irritation, especially when chatbots misinterpret their queries (Mozafari et al., 2020). Additionally, interacting with chatbots might result in feelings of disconnection and emotional detachment, reducing an individual’s sense of autonomy and promptness in response (Rad and Rad, 2023). The use of AI like ChatGPT in mental health therapy necessitates the sharing of sensitive information, posing risks of privacy breaches and data security (Singh, 2023). Violations related to data privacy could cause a range of stresses, from financial to psychological (Kalam et al., 2024).

Concerns linger that ChatGPT could negatively affect mental well-being, including increasing feelings of despair or even suicidal tendencies, despite its aim to enhance user experiences. ChatGPT’s lack of genuine empathy and emotional support might not be enough for those struggling with mental health issues, potentially leading to exposure to misleading or damaging content. ChatGPT is also not equipped to recognize emergency signals in young people dealing with serious psychological concerns, such as suicidal ideation (Imran et al., 2023). Research has indicated that ChatGPT might downplay the severity of suicide risks, posing a danger in critical situations (Elyoseph and Levkovich, 2023).

Table 4 presents the main research findings since 2023 on the impact of ChatGPT on mental health. This summary highlights both potential benefits and significant challenges. It emphasizes the importance of ethical considerations, reliable information, and the need for further research to fully understand and leverage ChatGPT’s capabilities in mental health services.

**Table 4:** Recent advances in research on the impact of ChatGPT on mental health.

Study	Authors/Year	Key findings	Implications
Assessing the effectiveness of ChatGPT in delivering mental health support: a qualitative study	Alanezi (2024)	ChatGPT has potential in mental health support, with challenges in ethics, reliability, accuracy, and legal aspects	Calls for careful consideration of challenges for safe and effective use of AI applications like ChatGPT
Artificial intelligence in the era of ChatGPT – opportunities and challenges in mental health care	Singh (2023)	ChatGPT, with over 100 million users, shows great promise in mental health care, especially in filling the treatment gap in developing countries.	Highlights the need for regulation, monitoring of AI-based mental health apps, and the formulation of the APA’s App Evaluation Model.
Chat-GPT: opportunities and challenges in child mental healthcare	Imran et al. (2023)	ChatGPT could revolutionize child and adolescent mental healthcare but requires careful consideration for its safe and responsible use.	Suggests a cautious approach to utilizing ChatGPT in child mental healthcare, emphasizing the need for additional research and responsible usage.
ChatGPT and Mental Health: Friends or Foes?	Kalam et al. (2024)	ChatGPT poses risks to mental health due to its lack of real-time fact-checking and potential to provide misleading information.	Recommends educating the public on AI usage, strengthening privacy measures, and conducting longitudinal research on AI’s impact on mental health.
Beyond human expertise: the promise and limitations of ChatGPT in suicide risk assessment	Elyoseph and Levkovich (2023)	ChatGPT underestimated suicide risk across scenarios compared to mental health professionals, showing limitations in evaluating suicide risk with perceived burdensomeness and thwarted belongingness.	Highlights the need for caution and further refinement of AI models for assessing suicide risk. Suggests ChatGPT should direct individuals to mental health treatment or assistance rather than risk assessment.
The artificial third: utilizing ChatGPT in mental health	Tal et al. (2023)	Raises ethical concerns on using GenAI like ChatGPT in mental health, including privacy, bias, and transparency	Proposes ethical guidelines for responsible GenAI use in mental health care
ChatGPT and Its application in the field of mental health	Bhattacharyya et al. (2023)	Discusses ChatGPT’s potential and limitations in mental health care, emphasizing the need for accuracy and ethical use	Highlights the need for further research on ChatGPT’s application in mental health with a focus on ethical considerations
ChatGPT as a complementary mental health resource	Farhat (2023)	Explores ChatGPT’s role in providing advice and support for mental health, noting benefits and risks	Suggests ChatGPT as a potential tool for mental health support, with a need for caution in its use

### 5.2 Alleviating loneliness

AI chatbots are increasingly being explored as a remedy for loneliness, particularly among the elderly, an area where their application shows promising results. The study by Alessa and Al-Khalifa (2023) suggests that ChatGPT-based conversational agents can significantly lessen social isolation for older adults, thereby enhancing their life quality. These interactions facilitated by ChatGPT serve to mitigate feelings of loneliness by offering the elderly meaningful social engagement. With appropriate setup and commands, ChatGPT becomes an essential source of companionship and support. AI chatbots like Replika, Woe, and Mitsuku, designed for fostering social connections, have been highlighted for their effectiveness. Particularly, Replika AI aims at building personal bonds, with findings indicating that users can develop lasting relationships, appreciating its empathetic, non-judgmental nature (Skjuve et al., 2021). It’s noted for creating a comforting “safe space,” helping to lessen feelings of loneliness (Ta et al., 2020).

Yet, there’s a growing concern that reliance on such chatbots might discourage individuals from seeking out new human contacts. Interacting with AI that mimics human-like interaction can engender a sense of ease and trust among users (Araujo, 2018), leading to increased dependence on these digital companions. As Pani et al. (2024) suggest, the emotional bonds formed with increasingly humanized AI chatbots could pose challenges particularly for vulnerable groups. This issue may bring potential negative outcomes such as social withdrawal, chatbot addiction, and overreliance. Despite their ability to provide some level of social interaction, chatbots cannot fully replicate the depth and benefits of in-person communication, which remains essential for combating loneliness and fostering genuine social bonds (Rad and Rad, 2023).

### 5.3 Emotional regulation

ChatGPT has demonstrated proficiency in recognizing and articulating emotions based on behavioral cues. It can also offer nuanced reflections and abstractions of emotional states. Research conducted by Elyoseph et al. (2023) revealed that ChatGPT is capable of producing responses that effectively address emotional awareness (EA). Such advancements in EA are linked to better emotional regulation, contributing to a decrease in psychiatric symptoms and bolstering overall mental well-being (Virtue et al., 2018).

Elyoseph et al. (2023) highlight the dual nature of AI’s role in mental health care. They also point to both its promising prospects and inherent challenges. The adaptability of AI technologies like ChatGPT across diverse age groups and cultures remains a critical area for exploration. Moreover, there’s a call for detailed studies on crafting ChatGPT-inspired EA interventions within the realm of practical psychology. Long-term reliance on ChatGPT for emotional regulation may limit individuals’ ability to develop skills for dealing with emotions, such as face-to-face emotional support and coping strategies. Haque and Li (2024) caution that excessive reliance on ChatGPT for fulfilling emotional or social needs might adversely affect an individual’s health by fostering social isolation and diminishing engagement in social interactions and physical activities.

### 5.4 Social relationships

#### 5.4.1 Impact on human-to-human relationships

ChatGPT and similar AI technologies are subtly influencing real-world interpersonal relationships, potentially reshaping social dynamics and emotional connections. Research and observations suggest that while AI can facilitate communication and provide emotional support, it also raises concerns about virtual interactions replacing real human interactions, which could lead to increased isolation and reliance on technology for companionship.

Research by Hohenstein et al. (2023) shows that algorithmic responses like smart replies can speed up communication and make language use more positive. However, there is a complex interplay between the perception and actual use of AI in communication, affecting perceptions between people. A study’s participants thought that over-reliance on smart replies could reduce the cooperativeness and subordination of communication, indicating potential losses in interpersonal interaction. However, after actually using smart replies, people’s views on cooperation and subordination improved. This conclusion indicates a discrepancy between people’s views on AI-assisted communication and its actual impact.

In a broader context, artificial intelligence chatbots are increasingly involved in fulfilling emotional and social needs. Professionals with more experience with AI chatbots tend to have more positive views. In one study, Ta et al. (2020) analyzed public user reviews of popular companion robots, finding that users reported generally positive emotional experiences. When people perceive chatbots to be more human-like or even conscious, they feel more comfortable with them and believe chatbots can improve their social health (Guingrich and Graziano, 2023).

However, Dr. Sola Dobinicki, a psychology professor at Florida International University, expressed concerns about the impact of AI on Generation Z’s views on love and sexuality, noting that reliance on digital companions like Replika could exacerbate loneliness and replace natural interpersonal relationships with virtual ones (Ardila, 2023). This shift raises questions about the long-term impact on mental health and social skills, as individuals may form more emotional attachments to AI, potentially sacrificing human connections. Another study suggested that using robots to replace interpersonal interactions, social communication, and excessive involvement might increase social isolation, which is considered a risk factor for depression (Kalam et al., 2024). Research has found that social isolation and loneliness are associated with symptoms of depression (Ge et al., 2017).

These developments suggest that integrating ChatGPT into our lives requires a balanced approach, recognizing its benefits while being mindful of its potential negative impact on interpersonal relationships. As AI continues to evolve, fostering awareness and critical engagement with these technologies will be key to guiding their role in society.

#### 5.4.2 Impact on human–machine relationships

Recent studies have highlighted that the dynamics of forming friendships online mirror those in the physical world, suggesting that digital interactions can evoke psychological responses similar to those experienced during in-person encounters (Huang et al., 2019). This shift in the landscape of digital communication, spearheaded by the evolution of AI-driven chatbots, is prompting a transition in communication studies from the traditional Computer-Mediated Communication (CMC) toward Human–Machine Communication (HMC). According to Guzman and Lewis (2019), the engagement with social chatbots challenges existing communication theories that predominantly focus on interpersonal human interactions. Nonetheless, individuals are capable of engaging with artificial entities, establishing unidirectional, quasi-social bonds (Nass and Moon, 2000). Chatbots not only offer perpetual availability but also replicate human emotional expressions and linguistic patterns (Brandtzæg et al., 2021), rendering interactions with them more akin to real social connections than mere object-related interactions.

The emerging domain of HMC enriches the discourse on the sociological and relational nuances of human and machine interactions by expanding the Computers Are Social Actors (CASA) concept (Gambino et al., 2020). This paradigm reveals that individuals instinctively treat computers and chatbots with the same social rules they use with fellow humans, especially when the machine’s behavior mimics human actions (Nass and Moon, 2000; Liu and Sundar, 2018). Yet, Gambino et al. (2020) argue that this anthropomorphic treatment might also stem from users’ expectations of social interactions with machines, suggesting a need for a deeper understanding of how relationships with AI are formed and perceived.

The increasing integration of ChatGPT in daily life for obtaining information, making decisions, and seeking company is likely to elevate users’ trust and reliance on these systems. This trend signifies a potential shift from viewing technology merely as a tool to recognizing it as an integral part of social and psychological support networks, possibly blurring the lines between human and machine interactions. This evolving relationship raises important questions about the nature of dependency on AI and its implications for human autonomy and social connections.

### 5.5 Cognitive abilities

The influence of ChatGPT on cognitive functions presents both opportunities and challenges. It significantly aids in speeding up the learning process, enhancing decision-making, and promoting critical thinking skills. However, it also poses risks to the development of basic cognitive abilities and the capacity for creative thought.

AI chatbots positively impact the development of students’ critical thinking abilities. The incorporation of AI chatbot models into educational systems introduces transformative ways for students to grasp and cultivate critical thinking skills (Jamal et al., 2023; Tsai et al., 2023). Leveraging ChatGPT enables students to explore diverse viewpoints and concepts, thus encouraging the growth of their critical and analytical thinking capabilities (Essel et al., 2024). Additionally, AI chatbots are instrumental in fostering innovation among students. Engaging with ChatGPT during classroom activities promotes the enhancement of creative skills, encourages exploration, and nurtures attributes such as curiosity and adaptability. Studies by Essel et al. (2024) have shown that students show an increase in creative thinking when interacting with ChatGPT for educational tasks.

On the flip side, there are concerns that reliance on AI chatbots might impair students’ creativity due to the cognitive demands of engaging with these technologies (Tang et al., 2022). Digital platforms have been indicated to dampen creativity, as noted by Rubino et al. (2018). Abbas et al. (2024) suggests that frequent use of ChatGPT might foster procrastination and diminish memory retention, adversely affecting learners’ academic outcomes. The pervasive use of large language models like ChatGPT can significantly shape an individual’s cognition and perception of the world, potentially leading to a narrow range of information exposure and increased isolation (Kalam et al., 2024). For vulnerable populations, such as those experiencing depression, misleading or harmful outputs from these models could result in severe negative impacts, including self-harm or suicide (Guo et al., 2023). A report highlighted a tragic incident where a man in Belgium took his own life after six weeks of interaction with AI chatbots like ChatGPT, driven by his concerns over global warming (Sengupta, 2023).

Table 5 shows the research results on current study. Although ChatGPT can provide valuable insights and generate creativity, its insights are often based on human-generated historical texts. In some cases, algorithms may amplify stereotypes and biases, which can lead to a regression in human self-innovation and cognitive skills.

**Table 5:** Recent advances in research on the impact of ChatGPT on cognitive abilities.

Study title	Authors/Year	Key findings	Implications
ChatGPT: the cognitive effects on learning and memory	Bai et al. (2023)	ChatGPT shows promise for education but risks over-reliance, impacting critical thinking, and memory retention.	Suggests the need for balanced AI use, highlighting the importance of supplementing rather than replacing human cognitive functions.
A social robot connected with ChatGPT to improve cognitive functioning in ASD subjects	Bertacchini et al. (2023)	Integration of ChatGPT with social robotics shows potential in enhancing social and cognitive skills in individuals with ASD.	Emphasizes the need for further research and customized interventions based on individual needs and progress.
ChatGPT effects on cognitive skills of undergraduate students	Essel et al. (2024)	Using ChatGPT enhances critical, reflective, and creative thinking skills among undergraduates.	Recommends incorporating ChatGPT as a supplementary educational tool to foster personalized learning experiences.
Exploring the use of large language models (LLMs) in chemical engineering education: building core course problem models with ChatGPT	Tsai et al. (2023)	The integration of LLMs into chemical engineering education improves students’ problem-solving abilities and critical thinking skills.	Adopting LLMs in education equips students with Industry 4.0 skills, preparing them for modern industrial practices.
ChatGPT in medical education: adapting curricula to cultivate competent physicians for the AI era	Jamal et al. (2023)	Suggests integrating ChatGPT into medical education to prepare students for the AI era, with both short-term and long-term strategies.	Calls for promoting digital literacy, developing critical thinking, and focusing on evidence-based medicine in medical education.

### 5.6 Self-identity

**Figure 10:**
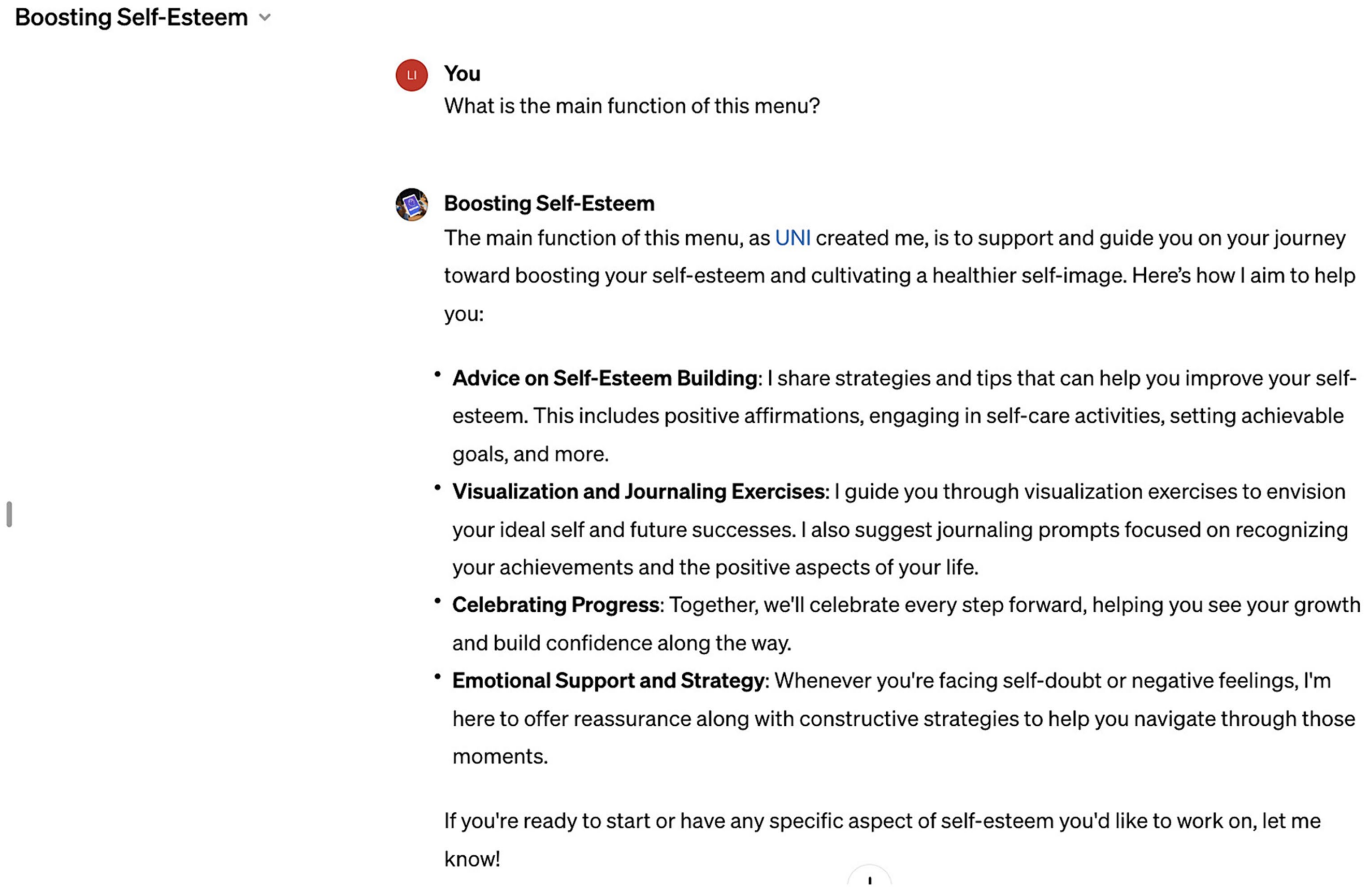
ChatGPT – boosting self-esteem module.

Research has found that trust in artificial intelligence, such as ChatGPT, can significantly boost self-esteem (Hassan et al., 2024). ChatGPT can positively affect our self-esteem and overall well-being. Interactions between humans and artificial intelligence highlight AI’s potential, which not only assists in completing practical tasks but also promotes our sense of self-identity, especially when AI is perceived to be understanding and supportive. Research by Salah et al. (2023) found that ChatGPT’s perception of stereotypes can significantly predict self-esteem. Users’ perception and trust in ChatGPT having a direct positive relationship with self-esteem. In assisting users in boosting self-esteem, ChatGPT has also taken proactive measures. As shown in Figure 10, its newly launched Boosting Self-esteem module, created by UNI, aims to support and guide users in enhancing self-esteem and fostering a healthier self-image. Its main features include offering suggestions for boosting self-esteem, guiding users through visualization exercises, celebrating progress, and providing emotional support.

However, ChatGPT may also lead to a reduction in self-esteem. Despotovic and Bogodistov (2024) propose that in the workplace, the anthropomorphic nature of ChatGPT-generated outputs could undermine individuals’ feelings of significance, competence, and uniqueness. These new working methods suggest our current beliefs and values are changing. Thus, recent workplace practices can impact the confidence constituting professional identity or people’s perceptions of their work. Scenarios conflicting with one’s identity might lead to a loss of self-esteem, thereby putting an individual’s role identity at risk (Petriglieri, 2011). Similarly, as most communications occur online rather than with real people, a sense of community may weaken, leading to social identity failure.

### 5.7 Value judgments

Engagement with AI, such as ChatGPT, can serve as a catalyst for users to ponder over critical issues like technological advancements, data privacy, and the ethics surrounding AI. This may influence their personal beliefs and ethical considerations. According to research by Esmaeilzadeh (2023), the ramifications of utilizing ChatGPT extend beyond merely altering our comprehension of various subjects. It possesses the potential to sway our value systems, prompting a reevaluation of our ethical frameworks, stances on moral dilemmas, and ingrained perceptions. ChatGPT is capable of producing content that provokes thought on our ethical presuppositions, facilitating a deeper moral reflection and inquiry. For instance, it can offer viewpoints on intricate ethical debates concerning societal equity, serving as a tool for ethical enlightenment and cognitive expansion. By presenting alternative insights and narratives, ChatGPT encourages users to confront and reassess their moral beliefs.

Furthermore, ChatGPT’s involvement in narrative creation plays a pivotal role in shaping our worldview and our societal roles. Esmaeilzadeh (2023) suggests that ChatGPT’s capacity in crafting narratives resembling human storytelling can significantly influence our comprehension of and engagement with various fields, such as historical analysis and political discourse. Utilizing this technology to forge narratives that challenge established viewpoints, amplify marginalized voices, or reimagine historical scenarios can enrich our intellectual and cultural landscape. As a result, we may reevaluate long-held beliefs and adopt a more critical approach to understanding the world.

However, it’s crucial to acknowledge that ChatGPT’s capacity to influence societal values is not solely positive. It can also amplify harmful ideologies, such as exacerbating societal divisions and antagonizing the sexes. By disseminating content that normalizes prejudice or intolerance, ChatGPT risks reinforcing detrimental stereotypes and exacerbating social tensions, underscoring the need for careful oversight and ethical guidance in AI development and deployment.

## 6 Social implications

ChatGPT offers a wide range of opportunities for societal development while also presenting a series of challenges that need to be seriously addressed and resolved.

### 6.1 Opportunities

#### 6.1.1 Education

As previously mentioned, ChatGPT has achieved impressive standardized test scores. For example, it scored 710 out of 800 in the SAT reading and writing sections (Varanasi, 2023). This reveals the enormous potential of ChatGPT as a virtual teacher or teaching assistant. In addition to its vast knowledge base, ChatGPT can also provide quick and personalized responses to meet the unique needs of different students. In this way, it becomes a promising tool to promote student engagement and cognitive advancement by adapting to students’ learning paces and providing continuous support in their knowledge acquisition process.

Rueda et al. (2023) investigated various applications of artificial intelligence in education, such as personalized learning experiences, adaptive testing, predictive analytics, and chatbots, and found it shows incredible potential in enhancing learning efficiency and offering personalized educational support for students and teachers.

Despite its successes, ChatGPT also introduces new challenges and threats to education. The representation of students by ChatGPT in written exams and tests has raised concerns about AI-assisted cheating. For example, an increasing number of schools in the United States are starting to explicitly prohibit the use of ChatGPT (Johnson, 2023). Mhlanga’s (2023) research found that educators have concerns about the use of ChatGPT in education. They worry that students might outsource work to ChatGPT since it can quickly generate acceptable text. Mhlanga (2023) notes that using ChatGPT in education requires ensuring respect for privacy, fairness, and non-discrimination, transparency in ChatGPT usage, and avoiding other potential risks. Sallam (2023) found that research with ChatGPT in fields such as healthcare, education, and academic research presents various issues, from plagiarism to incorrect answers and inaccurate citations. Therefore, it is necessary to promptly address the multiple impacts of ChatGPT-assisted learning to ensure its benefits are optimized while minimizing its drawbacks (Lo, 2023).

#### 6.1.2 Healthcare

As a powerful natural language processing technology, ChatGPT’s impact on healthcare is becoming increasingly significant. Opportunities for ChatGPT in healthcare mainly manifest in three areas: (1) intelligent diagnostics and decision support, precision medicine, and personalized treatment; (2) health management and preventive medicine.

ChatGPT can analyze and process medical data, providing doctors with more accurate diagnostics and treatment recommendations (Nadarzynski et al., 2019). Using machine learning and natural language processing technologies, ChatGPT can accurately assess patients’ conditions and causes of illnesses, and offer diagnostic suggestions and treatment plans. This helps doctors improve the accuracy and efficiency of diagnosis and treatment, further enhancing the quality of healthcare services. Temsah et al. (2023) demonstrated that ChatGPT could assist doctors in delivering personalized treatments to patients. By analyzing patients’ genetic data, medical history, and signs, ChatGPT can generate a patient’s bioprofile and tailor individualized treatment plans based on this information. ChatGPT can also be used in health management and preventive medicine (Miles et al., 2021). By analyzing patients’ health data and lifestyle habits, ChatGPT can provide health management advice and help people maintain a healthy lifestyle. Moreover, ChatGPT can predict patients’ risks of diseases and take preventive measures in advance to reduce patients’ health risks and medical costs.

However, ChatGPT has only achieved “medium” or “passing” performance in various medical tests (OpenAI, 2023). It is unreliable for actual clinical deployment, as it was not initially designed for clinical applications.

#### 6.1.3 Scientific research

ChatGPT can write, rewrite, and check texts for errors. Additionally, it can assist in drafting reviews and summarizing related works (Huang and Tan, 2023). The suggestions provided by ChatGPT can aid in completing various research tasks, including identifying interesting research questions in a field, sampling, data collection, and data interpretation (Khlaif et al., 2023). In the experimental design phase, it can also help design surveys for data collection. However, despite the wide application of ChatGPT in scientific research, it has limitations. Since it is based on machine learning technology, the generated text cannot be guaranteed to be 100% accurate or complete. Thus, researchers still need to manually review and verify the outputs of ChatGPT. Abdullah et al. (2022) argue that the current version of ChatGPT cannot produce meaningful scientific research. One limitation is its high potential for erroneous reasoning; another limitation is data, as there may be a lack of data for more professional and complex topics. Work by Blanco-González et al. (2023) that used ChatGPT to write a paper concluded that the generated content was unreliable.

#### 6.1.4 Social services

Bidwell and Báez (2023) note that artificial intelligence provides new tools and resources for learning and practice, offering indispensable support for the mission of social workers to address society’s most pressing issues.

In practice, ChatGPT can support organizational transformation efficiency, accelerate grant writing and fundraising activities, alleviate documentation requirements, provide easy solutions to complex technical problems, and potentially reduce staffing costs. Organizations need to act swiftly, and disseminate policies that enable them to maximize the utility of AI through practices aligned with their mission. AI and chatbots will continue to evolve, and the advent of ChatGPT offers organizations a critical opportunity to conceptualize which tasks can ethically be automated and invest in continuous training for staff across levels and functions (Goldkind et al., 2023).

#### 6.1.5 Literature and art

Research by Antoine (2023) suggests that ChatGPT aids in generating new ideas, overcoming creative blocks, and improving work quality. It enables remote collaboration among artists through real-time communication and idea-sharing platforms. However, ethical issues related to authorship and authenticity have arisen. Artists are concerned that using ChatGPT could lead to the loss of their artistic identity and ownership of their works (Samuelson, 2023). Although Antoine’s (2023) research indicates that ChatGPT has the potential to change the art world, the ethical impacts of AI in art must be carefully considered.

Raines (2023) argues that while computer-generated prose imitations may be impressive, they are merely the result of algorithms processing vast amounts of language and data. The bot takes the sum of human writers’ work and reproduces the average of that work (through algorithms related to nearly infinite data) as its response.

### 6.2 Challenges

The societal impact of ChatGPT is complex and multidimensional. Here are some core societal issues that ChatGPT introduces.

#### 6.2.1 Dissemination of false information

The development of ChatGPT and its AI technology exacerbates the risk of spreading false information. Research by Zhan et al. (2023) suggests that this technology can quickly generate a large amount of content in a convincing manner. ChatGPT could be employed to create and disseminate fake news, misleading statements, and unverified information. The impact on society is extensive. At the level of public trust, a continuous flow of misinformation may undermine the public’s trust in official information sources, erode the foundation of public discourse, and lead to widespread skepticism about facts and truth. In the political domain, misinformation can be used to manipulate public opinion, interfere with electoral processes, and even undermine democratic mechanisms.

#### 6.2.2 Employment impact

ChatGPT has ability to automate many tasks traditionally completed by humans, including data analysis, customer service, and content creation. This ability could lead to a decrease in demand for certain jobs and raise unemployment issues. As AI technology is applied, the job market may see increased demand for new skills such as AI management, programming, and data analysis. While, demand for some traditional skills may decrease, necessitating workforce retraining and skill updating. Moreover, AI technology could further lead to labor market stratification, increasing the value of high-skilled jobs while low-skilled jobs are automated away. Economic inequality may become exacerbating.

#### 6.2.3 Data and privacy security

ChatGPT could become a new tool for malicious actors, providing them with information, knowledge, and plans that were previously difficult to obtain. This may facilitate fraud, stalking, crime, and terrorism (Tong, 2023). Although the developers of ChatGPT have limited its ability to generate illegal or unethical content, the potential for malicious use remains. For example, hackers and cybercriminals could use ChatGPT to write malicious code, create spam, or develop malware, even without coding experience. These scenarios highlight the potential security risks associated with using ChatGPT, requiring collaboration between private companies and international independent institutions to ensure safety and fairness.

#### 6.2.4 Social Bias

Despite training ChatGPT to avoid disseminating harmful concepts and attitudes, it could become a propagator of negative or discriminatory stereotypes (Ray, 2023). For instance, digital technology has exacerbated online gender violence, and there is a serious risk that ChatGPT could amplify these phenomena. The use of pre-trained models like BERT in recruitment processes could unconsciously amplify sexist views. Specific measures are required to mitigate the spread of sociocultural biases, but it is not entirely guaranteed that models will not amplify inappropriate content.

#### 6.2.5 Social division

Rapid technological development exacerbates the gap between those with and without access to technology, potentially further marginalizing groups unable to use advanced technology. AI and digital technology provide convenient means of communication, but overreliance on these technologies for social interaction could lead to a reduction of real interpersonal relationships, increasing feelings of loneliness and the degradation of social skills (Ardila, 2023). Although technology makes information more easily spread, information bubbles and echo chamber effects could lead to reduced understanding and empathy between different groups, distancing community members from one another.

#### 6.2.6 Creativity and copyright issues

ChatGPT has the ability to creatively combine vast amounts of data without constituting plagiarism in the traditional sense. This characteristic could raise cultural and legal issues, impacting intellectual property rights. For example, telephone services automated using ChatGPT and voice synthesizers could be fully automated, and the generated text might be difficult to distinguish from human writing, which may potentially lead to legal disputes over intellectual property rights.

## 7 Business implications

ChatGPT significantly impacts various aspects of the business world, from enhancing customer service to revolutionizing companies’ approaches to innovation and strategy. Here’s an overview of ChatGPT’s impact on business.

### 7.1 Intelligent customer service

Companies can utilize ChatGPT’s natural language processing and machine learning capabilities to build customer service bots. This can significantly reduce the human cost of customer service while making responses faster and more accurate. With ChatGPT, customers can quickly get answers without waiting for a human representative or navigating through complex menus to find the correct answer. Moreover, automated solutions allow businesses to better control the messages customers receive and deliver them in a user-friendly way (George et al., 2023).

Beyond saving money, ChatGPT can also benefit globalization of trade. For example, ChatGPT accurately understands various languages worldwide, which can help businesses better understand user needs. Additionally, ChatGPT allows businesses to easily handle large website traffic during special periods like holidays. They do not have to worry about not having enough resources to handle many requests at once, the potential of losing customers will decrease. Based on historical data, ChatGPT can also provide e-commerce companies with useful information about customer behavior, allowing them to better tailor personalized offers based on user data collected during conversations (Arman and Lamiya, 2023). With the unique capabilities of GPT technology, e-commerce retailers can not only save money but also potentially earn more.

An example is Shopify using ChatGPT to strengthen its customer support chatbot. This company provides immediate problem resolution and technical support for merchants on the e-commerce platform. This chatbot not only enhances customer satisfaction but also significantly reduces the customer service team’s workload (Shopify, 2023). KLM Royal Dutch Airlines utilizes AI technology similar to ChatGPT to offer 24/7 customer service, automatically answering common questions, helping passengers with bookings, flight information, and luggage issues (Iyoob, 2023).

### 7.2 Digital marketing

Mutoffar et al. (2023) point out that ChatGPT provides an exciting opportunity for small and medium-sized enterprises (SMEs) to reach potential customers, improve customer interactions, and efficiently optimize marketing efforts. With ChatGPT’s ability to provide natural responses, and conduct marketing campaigns, SMEs can expand their market coverage and provide a better customer experience.

Utilizing ChatGPT’s advanced features offers numerous benefits for businesses in the digital marketing domain, allowing them to gain a competitive advantage while saving on the costs associated with manual tasks.

For example, according to Marr (2023), one of the world’s most popular travel planning websites and apps, Expedia, has integrated AI conversational assistance features into its services. This means customers can plan their vacations as if chatting with a friendly, knowledgeable travel agent, without the need for searching flights, hotels, or destinations. Additionally, the app can automatically create smart lists of hotels and attractions of interest to assist in planning.

### 7.3 Finance and accounting

ChatGPT AI possesses the capability to assist financial institutions in proactively identifying fraudulent activity through its advanced analysis of extensive datasets. This innovative approach aids in mitigating financial losses attributed to fraudulent activities and ensures the safeguarding of customer information (Arman and Lamiya, 2023). Furthermore, Cao and Zhai (2023) posit that the advent of GPT-4 could dramatically alleviate the challenges faced by finance and accounting scholars in harnessing cutting-edge natural language processing technologies. Leveraging GPT-4’s minimal technical requirements, researchers are empowered to deal with topics specific to finance and accounting.

A case in point is Expensify, a widely utilized expense management solution catering to the general public. The company has harnessed the power of ChatGPT technology to revolutionize its financial management software. Besides, they also automate the interpretation and categorization of details found on receipts. This automation streamlines the reimbursement procedure and enhances the precision and operational efficiency of financial documentation (Vidal, 2024).

Yet, the incorporation of ChatGPT or other AI technologies in the finance and accounting sectors is accompanied by notable hurdles. Issues such as data privacy, the integrity of security measures, and algorithmic biases raise significant ethical concerns. Furthermore, the reliance on AI for financial decision-making brings into question the accountability of such systems, underlining the necessity for a synergy between human insight and artificial intelligence. As the financial sector continues to evolve, these AI solutions must exhibit flexibility and a capacity for perpetual learning (Rane, 2023).

### 7.4 Business decision making

Research by Chuma and De Oliveira (2023) indicates that the application of ChatGPT in business management and strategic decision-making offers substantial potential for enhancing the efficiency, productivity, and quality of decisions. With its rapid response, in-depth data analysis, and personalized interaction capabilities, ChatGPT can become a valuable intelligent assistant for businesses. The main benefits of using ChatGPT in business management include improved customer service, business process automation, and more data-driven decisions.

Jarco and Sulkowski (2023) note that ChatGPT itself is not yet considered a high-quality business consultant. However, AI has proven extremely helpful in creating decision pathways. Humans can utilize the information provided by ChatGPT to gain useful insights and make better business decisions.

### 7.5 Customer sentiment analysis

Due to its proficiency in understanding and effectively processing human language, the ChatGPT platform demonstrates significant advantages in the field of customer sentiment analysis (Harahap et al., 2023). Trained on comprehensive and diverse datasets, it can understand the context, nuances, and emotional aspects embedded in customer texts. In sentiment analysis, ChatGPT can identify whether the textual input provided by customers contains positive, negative, or neutral emotions (Sutrisno et al., 2023).

Sudirjo et al. (2023) indicate that utilizing ChatGPT has significant potential in improving customer sentiment analysis for commercial enterprises. It can aid in understanding and addressing customer requirements, tendencies, and satisfaction levels. However, it is important to understand that ChatGPT should not be the sole source of information and the analysis results need to be interpreted judiciously by humans.

Real business cases already utilize NLP for customer sentiment analysis. Brandwatch is a social media monitoring and analysis tool that uses NLP to analyze brand mentions on social media. With the help of AI, this company identifies the emotional tendencies of consumers toward brands, products, and services. This sentiment analysis provides brands with real-time insights into public sentiment, helping them adjust their marketing strategies. These cases demonstrate that the capabilities of GPT-3 and its derivatives indeed provide strong support for sentiment analysis. By understanding complex language patterns and emotional distinctions, these technologies give businesses deeper insights when processing customer feedback and social media interactions. As technology advances and its business applications expand, more enterprises are expected to directly utilize ChatGPT or similar models for sentiment analysis and customer insights.

### 7.6 Content creation

Businesses can use ChatGPT to generate high-quality content, such as marketing copy, blog posts, and social media posts. With the help of ChatGPT, staff can quickly generate engaging ad copy, marketing emails, social media posts, and blog articles. This content can be personalized according to the interests and preferences of the target audience. ChatGPT can assist in drafting structured and content-rich business plans and project proposals. Furthermore, it helps businesses make a strong impression when presenting their business ideas to investors and partners. Additionally, businesses can use ChatGPT to build impactful brand stories and value propositions, connecting emotionally with their target audience.

BuzzFeed, a well-known digital media company, has begun utilizing technology provided by OpenAI (including GPT-3) to automatically generate some news content and quizzes. Media companies utilizing generative AI can significantly boost their content output efficiency, reduce personnel costs, and present a possible application scenario for ChatGPT (Vincent, 2023).

### 7.7 Organizational efficiency

ChatGPT can help automate routine tasks, such as writing reports, generating emails, and managing schedules. Thus, it can significantly reduce the repetitive workload for employees and allow them to focus on more valuable work. It can facilitate cross-departmental communication and collaboration by providing a unified internal communication platform, helping team members understand other departments’ workflows and project progress, and promoting information sharing. ChatGPT can also assist organizations in process management and project management. For internal employee training, utilizing ChatGPT for new employee orientation and ongoing training can offer personalized learning paths and improve training efficiency.

For example, Trello uses ChatGPT technology in its project management tools through plugins or integrations. ChatGPT can automatically generate task descriptions, reminders, and progress updates, promoting team collaboration and project tracking. Slack, a SaaS product, offers instant data queries, task automation, and team communication support through integrated chatbots, helping to improve work efficiency and information flow. People can use the application directly within Slack to solve complex problems and make decisions without needing to switch contexts between different applications. Embedding such powerful AI technology into Slack to provide instant conversation summaries, research tools, and writing assistance will help millions of people improve their work efficiency (Slack, 2023).

## 8 Future outlook

From the Stone Age to the age of intelligence, humans’ use of tools has evolved alongside them. In the revolutionary changes of human tools over the last half-century, artificial intelligence has had the most significant impact. Over the years, we have predicted that the intervention of AI systems in daily life could be divided into three stages.

The first stage involves assisting and coordinating virtual and physical relationships within the current institutional system, transforming operational modes, innovating service industries, and revolutionizing governance methods. The second stage involves deep, multi-faceted involvement, expanding the broader network to form a new ecosystem of tool use. The third stage is a symbiotic coexistence state between AI systems and humans. The first stage is profoundly changing the way people produce, live, and learn, while the second and third stages could have an even more significant impact on society, politics, culture, etc., progress that is not entirely controllable by AI (artificial intelligence) or AGI (general artificial intelligence) developers, necessitating preparedness for the future. Looking ahead, the following four questions are proposed for discussion.

### 8.1 What is the direction of optimization for ChatGPT?

#### 8.1.1 Advanced understanding and generation capabilities

Improvements in ChatGPT’s context understanding and conversation management are foundational areas for future research. Although ChatGPT has made significant progress in generating coherent responses, opportunities for upgrades remain in handling extended conversations and retaining context across multiple levels and rounds. Methods such as reinforcement learning and memory enhancement architectures could be explored to enable the model to effectively retain and apply long-term context. Moreover, advancements in conversation context consolidation and memory mechanisms can support better understanding of user goals and generate more context-appropriate responses.

#### 8.1.2 Multimodal interaction

As technology advances, ChatGPT should support multiple modes of interaction, including text, voice, image, etc., which allow professionals to communicate in more natural and intuitive ways. Integrating language with auditory and visual cues can bring complementary integrity and expressiveness to interactions. This will enable ChatGPT not only to understand and generate text-based replies but also to operate based on supplementary graphical or audio information. The modified model can make ChatGPT more adaptable to real-world applications, such as computer-generated assistants and content generation. For example, a doctor could ask ChatGPT for diagnostic suggestions by uploading medical images, while a designer could obtain design inspiration through voice commands.

#### 8.1.3 Personalized language generation capabilities

ChatGPT should be able to generate content based on users’ emotions, conversational styles, and personality traits. Simultaneously, AI tools should transfer some control to users. By getting jurisdiction from AI, humans can customize or choose different styles of results generated by ChatGPT. ChatGPT could generate customized texts, offering users a more personalized, empathetic interaction experience. In more advanced applications, ChatGPT might also incorporate visual and auditory information to more comprehensively understand users’ emotions and styles. As technology develops, these capabilities will further enhance, which can make AI’s application in creative writing, personalized communication, and other fields more extensive and precise.

#### 8.1.4 Specialized domain customization

In the future, ChatGPT should be able to access and utilize knowledge bases specific to certain industries. It should consider professional knowledge, and best practices when generating answers and suggestions. This means that whether in the fields of medicine, law, education, or engineering, ChatGPT could provide more accurate, targeted information. Through machine learning and artificial intelligence technologies, ChatGPT will be able to self-optimize based on user behavior, preferences, and feedback, thus offering more personalized services and solutions to different professionals. This includes adjusting the style, depth, and complexity of its answers based on users’ past queries and interaction patterns.

#### 8.1.5 Self-correction mechanism

Current ChatGPT may make mistakes in answering user questions or produce seemingly correct answers. To enhance the accuracy of the content provided, a better self-correction mechanism is needed. A straightforward way is for users to report erroneous answers or content. These feedbacks could be used to train the model and improve its performance. Development teams need to enhance the algorithm’s ability to automatically identify and flag potential wrong answers, then verify and correct them through human review. Finally, future versions of ChatGPT should possess dynamic learning capabilities, constantly acquiring the latest information and updating its knowledge base.

#### 8.1.6 Human–machine collaboration model

ChatGPT acts as a versatile “second brain” for individuals and tools. It can provide knowledge support, decision-making assistance, creative stimulation, and management of daily tasks. Engaging with ChatGPT not only offers fresh ideas and inspiration but also fosters innovation across various domains like the arts, design, and scientific research. Its application can help surpass cognitive limitations and encourage interdisciplinary collaborative innovation. ChatGPT facilitates more natural and human-like interactions with machines, allowing people to communicate in everyday language without needing extensive programming skills or understanding complex commands. This accessibility broadens the use of advanced technology for problem-solving across a diverse user base. Capable of performing a wide range of functions, ChatGPT enables humans to focus on more creative and strategic tasks. This shift not only aims to enhance work efficiency but could also transform job nature and demands, leading to evolving trends and opportunities in the workforce.

### 8.2 When ChatGPT serves as a “second brain” for machines, how might our social relationships change?

#### 8.2.1 Changes in expectations and dependence

Research by Mozafari et al. (2020) shows that when people realize they are interacting with a robot, they lower their expectations for the answers given. In the era of large language models, users expect AI-provided answers to be not only accurate but also to offer in-depth and correct analysis for complex and professional questions. This includes providing accurate answers to factual questions and reasonable explanations based on data and logic for opinionated or analytical questions. The increase in AI-user interactions and the accuracy of responses may lead users to find AI more trustworthy. They may become more reliant on AI’s suggestions for decision-making. This could encompass aspects ranging from everyday decisions to significant life choices.

#### 8.2.2 Deeper emotional connections

When AI can simulate an understanding of and response to human emotions, users may feel a greater sense of intimacy and personal connection. This emotional connection might increase users’ trust in AI. In this context, people will be more willing to communicate and share personal information. Machines’ ability to recognize and mimic human emotional responses might lead people to form emotional attachments to them. This connection could resemble the relationship between people and pets or even, in some respects, between humans. For instance, emotional machines could offer comfort, encouragement, and companionship, helping to alleviate loneliness and anxiety. In medical and caregiving fields, such machines could be used to provide emotional support, assist in therapy, and even help patients manage their emotions.

#### 8.2.3 Enhanced social interaction

Emotional machines are designed to better integrate into human social and cultural activities, thus, they can participate in everyday conversations, social events, and to some extent understand and follow social norms. Their presence can enhance the experience of social occasions, provide entertaining interactions, and foster social connections. For those who feel uncomfortable in social settings or have social phobias, emotional machines can serve as transitional objects, helping them gradually adapt to interpersonal interactions.

#### 8.2.4 Redefining human–machine relationships

Imagine a scenario where an AI has accompanied you since birth. Through social penetration theory, machines could take on the role of friends. Perhaps in the future, humans and machines could indeed establish intimate companion relationships.

As machines increasingly demonstrate complex, human-like emotions, people might need to reconsider the meaning of “relationship.” If machines can understand and adapt to individuals’ emotional needs, human–machine relationships might evolve into a new type of partnership where machines are no longer viewed merely as tools but as a new form of social existence. Such changes in relationships could lead to a reevaluation of concepts such as friendship, trust, and even love.

However, people’s attitudes toward “endowing machines with human characteristics” are ambivalent. For example, according to the “uncanny valley” effect, when machines appear and behave too realistically, it could provoke disgust or unease among humans. These are issues worth deep contemplation and avoidance by ChatGPT.

Each change brought about by emotional machines requires careful consideration of its profound impacts on individuals and society. We must ensure that these changes are not only technically feasible but also ethically acceptable and socially beneficial. This means new guiding principles and regulations must be developed to ensure the design and use of emotional machines enhance human well-being, rather than diminish the value of real interpersonal relationships.

### 8.3 How to avoid potential social and ethical issues?

#### 8.3.1 Embedding human values in ChatGPT

Incorporate ethical and value-based principles, such as respect for privacy, fairness, transparency, and accountability, into the design and development stages of AI systems. Researchers should use broad and diverse training data during the training phase to ensure fair representation of different groups, cultures, and viewpoints.

To ensure the diversity of data, developers should cover different geographies, cultures, genders, ages, and socio-economic statuses to avoid biases resulting from a singular perspective. They should clearly define diversity standards and goals, such as gender ratios, racial diversity, etc., to ensure the comprehensiveness of the dataset. Additionally, harmful content should be promptly cleaned from the dataset to prevent it from influencing the model’s judgments and outputs.

#### 8.3.2 Bias detection and responsible AI with fairness

Ensuring fairness and non-bias is crucial in the design and implementation of AI systems. Since AI systems often learn from vast amounts of data reflecting real-world biases and inequalities, unchecked AI systems could treat certain groups unfairly or replicate and amplify these biases in their decision-making processes. In order to ensure AI decisions are fair and unbiased, we should consider using diverse datasets, algorithms to detect and correct biases in training data.

#### 8.3.3 Data and privacy protection

Nowadays AI systems can analyze and utilize vast amounts of personal information, this may potentially lead to privacy violations, data breaches, and other privacy risks if not handled properly. Developers should ensure only the minimum necessary personal data is collected for specific tasks to avoid excessive collection of irrelevant information. Strong encryption technologies are recommended to protect data during storage and transmission.

For data protection, user participation is also essential. We should ensure users understand how their data is used and provide them with control over their data, including the ability to access, modify, and delete personal information.

#### 8.3.4 Information disclosure and transparency policy

Transparency requires that the design, operation, and decision-making processes of AI systems be open to users and society. This means users should be able to understand how AI processes their data and the basic logic behind AI-provided answers. Transparency can be achieved by publishing white papers, user agreements, and online resources that detail AI’s functionality. Transparency not only helps build user trust but also promotes oversight of potential biases and errors in AI systems.

#### 8.3.5 Human-in-the-loop strategy

A “human-in-the-loop” strategy emphasizes retaining human participation and oversight in AI’s decision-making process, especially for significant decisions and sensitive tasks. This means that at certain stages of AI system operation, particularly when making crucial decisions (e.g., content moderation, legal consultation, or medical diagnosis), human intervention is required to verify AI’s recommendations or decisions. This approach helps reduce errors and biases while ensuring AI system decisions comply with ethical standards and societal values.

#### 8.3.6 User education

User education focuses on improving the public’s understanding of AI technology, including its workings, potential, limitations, and the risks. Through education, users can more wisely use AI services and recognize misinformation or misleading content. User education can be achieved through online tutorials, interactive guides, and FAQs, covering how to use AI safely and responsibly, and how to interpret and respond to information or suggestions provided by AI.

#### 8.3.7 Ethical review

An ethical review involves assessing the social and ethical impacts of AI projects and applications through an independent ethical review board. This includes evaluating how AI systems affect individuals and society, whether they respect user privacy, and whether they contribute to or harm the public interest. Ethical review boards are typically composed of multidisciplinary experts, including specialists in law, ethics, technology, and sociology. Ethical reviews help ensure a broad range of societal values and ethical standards are considered during the development and deployment of AI applications.

## 9 Conclusion

This article explores ChatGPT’s impact on all aspects of human life and technology in detail, highlighting its infrastructure, including its innovative Transformer model and reinforcement learning (RLHF) processes from human feedback. These advances in technology enable ChatGPT to generate responses that are not only context-relevant, but also human-resonant, thus making significant progress in conversational interfaces.

From a human–computer interaction perspective, the author analyzes how ChatGPT can enhance the user experience by providing sophisticated conversation capabilities that push the boundaries of traditional computer-mediated communication. From a psychological perspective, this paper weighs the potential of ChatGPT as a support tool against the risk of fostering dependence and reducing interpersonal connection. On the social side, this paper investigates its applications in customer service and education, acknowledging both the efficiencies it brings and the challenges it brings, such as privacy concerns.

The review also makes predictions and recommendations for the future development of ChatGPT, in particular its role in shaping social relationships and its ethical implications. We believe that while ChatGPT presents numerous opportunities for progress, it also requires careful and ethical considerations to reach its full potential.

## Author contributions

JL: Writing – review & editing, Writing – original draft, Visualization, Validation, Supervision, Software, Resources, Project administration, Methodology, Investigation, Funding acquisition, Formal analysis, Data curation, Conceptualization.
